# Defining G protein-coupled receptor peptide ligand expressomes and signalomes in human and mouse islets

**DOI:** 10.1007/s00018-018-2778-z

**Published:** 2018-02-17

**Authors:** Patricio Atanes, Inmaculada Ruz-Maldonado, Ross Hawkes, Bo Liu, Min Zhao, Guo Cai Huang, Israa Mohammed Al-Amily, Albert Salehi, Stefan Amisten, Shanta J. Persaud

**Affiliations:** 1 Department of Diabetes, Faculty of Life Sciences and Medicine, King’s College London, London, UK; 2 Division of Islet Cell Physiology, Department of Clinical Science, SUS, University of Lund, Malmö, Sweden; 3 Department of Diabetes, Faculty of Life Sciences and Medicine, King’s College London, London, SE1 1UL UK

**Keywords:** GPCRs, Islets of Langerhans, Peptide ligands, PYY, Type 2 diabetes

## Abstract

**Introduction:**

Islets synthesise and secrete numerous peptides, some of which are known to be important regulators of islet function and glucose homeostasis. In this study, we quantified mRNAs encoding all peptide ligands of islet G protein-coupled receptors (GPCRs) in isolated human and mouse islets and carried out in vitro islet hormone secretion studies to provide functional confirmation for the species-specific role of peptide YY (PYY) in mouse islets.

**Materials and methods:**

GPCR peptide ligand mRNAs in human and mouse islets were quantified by quantitative real-time PCR relative to the reference genes ACTB, GAPDH, PPIA, TBP and TFRC. The pathways connecting GPCR peptide ligands with their receptors were identified by manual searches in the PubMed, IUPHAR and Ingenuity databases. Distribution of PYY protein in mouse and human islets was determined by immunohistochemistry. Insulin, glucagon and somatostatin secretion from islets was measured by radioimmunoassay.

**Results:**

We have quantified GPCR peptide ligand mRNA expression in human and mouse islets and created specific signalomes mapping the pathways by which islet peptide ligands regulate human and mouse GPCR signalling. We also identified species-specific islet expression of several GPCR ligands. In particular, PYY mRNA levels were ~ 40,000-fold higher in mouse than human islets, suggesting a more important role of locally secreted Pyy in mouse islets. This was confirmed by IHC and functional experiments measuring insulin, glucagon and somatostatin secretion.

**Discussion:**

The detailed human and mouse islet GPCR peptide ligand atlases will allow accurate translation of mouse islet functional studies for the identification of GPCR/peptide signalling pathways relevant for human physiology, which may lead to novel treatment modalities of diabetes and metabolic disease.

**Electronic supplementary material:**

The online version of this article (10.1007/s00018-018-2778-z) contains supplementary material, which is available to authorized users.

## Introduction

G protein-coupled receptors (GPCRs) play important roles in regulating secretion of the islet-derived gluco-regulatory hormones insulin, glucagon and somatostatin, and more than one-third of all GPCRs expressed by human islets are activated by ligands that are either proteins or polypeptides [1]. Many proteins and peptides that act as ligands of islet GPCRs are expressed by the islet cells themselves [1], while others are secreted from non-islet cell types and transported to the islet microenvironment by the systemic circulation. Examples of locally produced peptide ligands of islet GPCRs include glucagon and somatostatin, which act in a paracrine function to regulate secretion of islet hormones from neighbouring cells [2]. In addition, collagen III and IV, which are components of the extracellular matrix that supports islet cells, interact with the islet-expressed GPCRs, GPR56 and GPR126 [3–5]. GPCR peptide ligands that are delivered to islets in the circulation include the incretin hormones GLP-1 and GIP, which are secreted by specialised enteroendocrine cells in the gastrointestinal tract [6], and it has recently been reported that the cardiomyocyte-derived atrial natriuretic peptide stimulates insulin secretion via activation of β-cell guanylate cyclase-coupled GC-A receptors [7].

It is clear that peptide and protein (collectively referred to as ‘peptides’) GPCR ligands are important regulators of islet function and the maintenance of glucose homeostasis [1]. The majority of studies investigating the roles of islet-derived peptide ligands acting at GPCRs to regulate islet function have been carried out in vitro using mouse islets or in vivo using mouse models of metabolic disease. However, to date no systematic studies have been carried out to investigate how effectively observations made in these mouse models are translatable to the human setting. The notion that there might be significant differences in GPCR/peptide signalling pathways in mouse and man is supported by the observations that in some respects rodent and human islets show distinctive gene expression profiles [8–12]. In particular, we have recently reported that although the majority of GPCR mRNAs are common to mouse and human islets, some that are expressed by mouse islets are absent or expressed at only trace levels in human islets, and studies on these receptors in rodent islets will not be translatable to the human context [11]. Moreover, mouse and human islets have distinct architecture such that mouse islet function relies mainly on homogenous cellular communications between β-cells, with a simple framework distribution of cell subpopulations, while human islets are formed by heterogeneous cell populations [13, 14]. The increased contacts between adjacent endocrine cell populations in human islets suggest that the intra-islet paracrine signalling is likely to be more complicated than in rodent islets, in terms of signalling molecules interacting with their respective receptors and triggering autocrine or paracrine regulatory circuits [15].

In the current study, mRNA expression of all peptide ligands of islet GPCRs has been quantified and used to generate “signalome” atlases mapping the pathways by which peptide ligands regulate human and mouse islet GPCR signalling. These atlases can be used to identify the extent to which GPCR peptide ligand gene expression and GPCR/peptide signalling pathways are common to mouse and human islets, which will facilitate the translation of experimental results obtained using mouse islets and mouse models of metabolic disease to the human islet context.

## Materials and methods

### Reagents

TaqMan RT-PCR kits were from Thermo Fisher Scientific (Loughborough, UK) and QuantiTect SYBR Green qPCR kits with QuantiTect qPCR assays were from Qiagen Ltd. (Manchester, UK). The glucagon antibody was from Sigma-Aldrich (Dorset, UK), insulin antibody from DAKO UK Ltd. (Ely, UK) and the anti-PYY and somatostatin antibodies were from Abcam plc (Cambridge, UK). Anti-rabbit, anti-guinea pig, anti-rat and anti-mouse secondary antibodies were from Jackson ImmunoResearch (Suffolk, UK). [^125^I]-glucagon was from Millipore (UK) Limited (Watford, UK) and somatostatin EURIA was from Euro Diagnostica AB (Malmö, Sweden). All other chemicals were from Sigma-Aldrich or Thermo Fisher Scientific.

### Islet isolation and culture

Islets were isolated from 10 to 12 weeks old male mice of the inbred C57BL/6 (C57; Charles River) and outbred Crl:CD1 (ICR) mice (Charles River) strains by collagenase digestion of the exocrine pancreas [16]. All animal procedures were carried out in accordance with the UK Home Office Animals (Scientific Procedures) Act 1986. Human islets were isolated from heart-beating non-diabetic donors as previously described [17], with appropriate ethical approval for research use. Isolated mouse and human islets were maintained in culture overnight (mouse: RPMI 1640; human: CMRL) at 37 °C, 5% CO_2_ before experimental use.

### RNA extraction and quantitative real-time PCR

Total RNA was isolated from human islets and islets isolated from C57 and ICR mice using a modified TRIzol protocol followed by RNA clean-up on Qiagen RNEasy MinElute columns [18], and total RNA was converted into cDNA using the TaqMan RT-PCR kit [19]. Extensive manual searches in PubMed (http://www.ncbi.nlm.nih.gov/pubmed), IUPHAR (http://www.iuphar-db.org/) and Ingenuity Pathways Analysis (http://www.qiagen.com) were used to identify 159 human and 147 mouse orthologue genes encoding > 200 mature peptides known to act as agonists at human and mouse GPCRs. Proteases acting as activating ligands at protease-activated receptors (PAR1–4) were not included in this study, as they are not true ligands of the PARs. Human and mouse GPCR peptide ligand genes were quantified by real-time qPCR using Qiagen QuantiTect SYBR Green mouse or human primer assays, as described in detail elsewhere [20]. All human and mouse peptide ligand gene expression data were normalised relative to expression of the average of five reference genes (ACTB, GAPDH, PPIA, TBP and TFRC) amplified in the same samples. All qPCR amplification products were analysed by electrophoresis on 2% agarose gels to confirm that each amplification product matched the theoretical qPCR product size for the primer pairs used. All peptide ligand and reference gene primer efficiency (E) values [21] were in the range of 1.85–2.15 and template cDNAs were diluted in such a way that all quantified genes returned Ct values < 30. Genes expressing < 0.001% the mRNA expression of ACTB, GAPDH, PPIA, TBP and TFRC were considered to be present only at trace level, as their expression was outside of the lower end of linear quantification of the QuantiTect primer assays. Enrichment of GPCR peptide ligand mRNA expression in human islets was calculated using this formula:

$$ {\text{Enrichment of GPCR peptide ligand expression}}\, = \,{\text{Human islet GPCR peptide ligand expression relative to reference genes}}/{\text{Mean mouse islet GPCR peptide ligand expression relative to reference genes in ICR and C57 mouse islets}}. $$

For enrichment of GPCR peptide ligand expression in mouse islets, the inverse of the above formula was used.

### Construction of the human and mouse islet GPCR/peptide ligand signalome atlases

The pathways through which peptide ligands interacted with islet GPCRs were identified by manual searches in the PubMed, IUPHAR and Ingenuity databases and separate GPCR/peptide ligand pathways were constructed based on the mRNA expression of the peptide ligands and their receptors in human and mouse (ICR and C57) islets. The expression profiles of islet GPCRs that are activated by peptide ligands were extracted from our recent study that focused on defining expression of all GPCRs by human and mouse islets [11]. Those GPCRs and GPCR peptide ligands that had confirmed islet mRNA expression at trace level or above were included in the human and mouse islet signalome atlases described in this paper. Peptide ligand genes that were absent in islets, but which are known to be expressed in other tissues, were not included in the atlases but GPCRs that were not expressed by human and mouse islets were included, to indicate that islet-derived peptide ligands have the potential to act at remote GPCRs.

### Immunohistochemistry

Expression of PYY by cells within 5-μm sections of mouse and human pancreas was determined using a rabbit-derived antibody directed against PYY (1:50) and an anti-rabbit AlexaFluor^®^ 488 secondary antibody (1:200). PYY localisation in islet cells was probed by co-staining with guinea pig anti-insulin (1:200), mouse anti-glucagon (1:100) and rat anti-somatostatin (1:50) antibodies and species-specific Alexa Fluor^®^ 594 secondary antibodies (all at 1:200). Negative control sections were probed with AlexaFluor secondary antibodies in the absence of primary antibodies. Immunostained pancreas sections were visualised under a TE2000 fluorescent microscope (Nikon, Kanagawa, Japan) and analysed using Image J software (https://imagej.net/). The proportion of cells expressing PYY in each islet cell subpopulation was calculated by dividing the mean number of PYY-positive cells by the number of β-, α- or δ-cells per islet.

### Quantification of insulin, glucagon and somatostatin secretion from mouse and human islets

Groups of ten mouse and human islets were incubated for 1 h in a physiological salt solution [22] in the absence or presence of the high affinity NPY1R antagonist, BIBO and insulin, glucagon and somatostatin in the supernatants were quantified by competitive radioimmunoassay, essentially as described elsewhere [23–25].

### Statistical analysis

GraphPad Prism 5.0 (GraphPad Software, Inc.) was used for statistical analyses. Data are presented as mean ± SEM and were analysed using ANalysis Of VAriance (ANOVA). Statistical significance was set at *p* values of < 0.05 (*), < 0.01 (**), < 0.001 (***), considering *p* < 0.05 as statistically significant.

## Results

### Expression of GPCR peptide ligand mRNAs in human and mouse islets

**Fig. 1:**
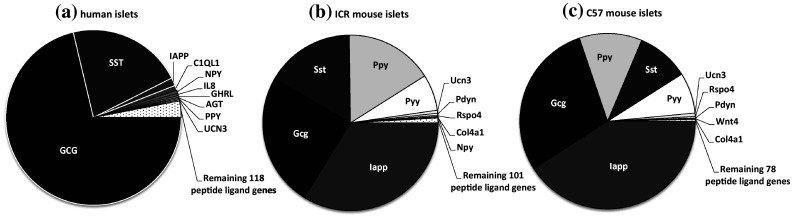
Summary of the mean relative expression of the ten most abundant GPCR peptide ligand genes in human islets (**a**) and ICR (**b**) and C57 (**c**) mouse islets. Data for each peptide are presented as mean % of the mRNA expression of all GPCR peptide ligands in each islet type

**Fig. 2:**
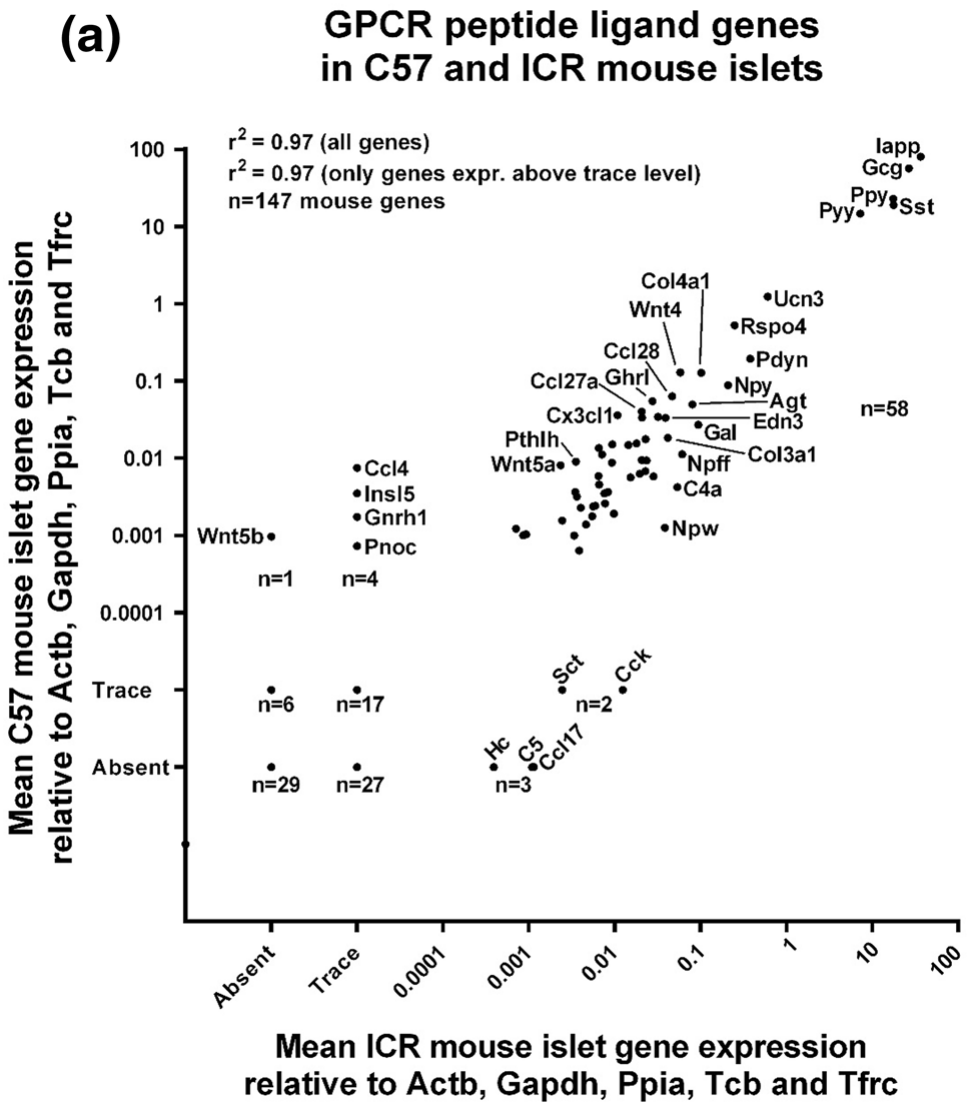

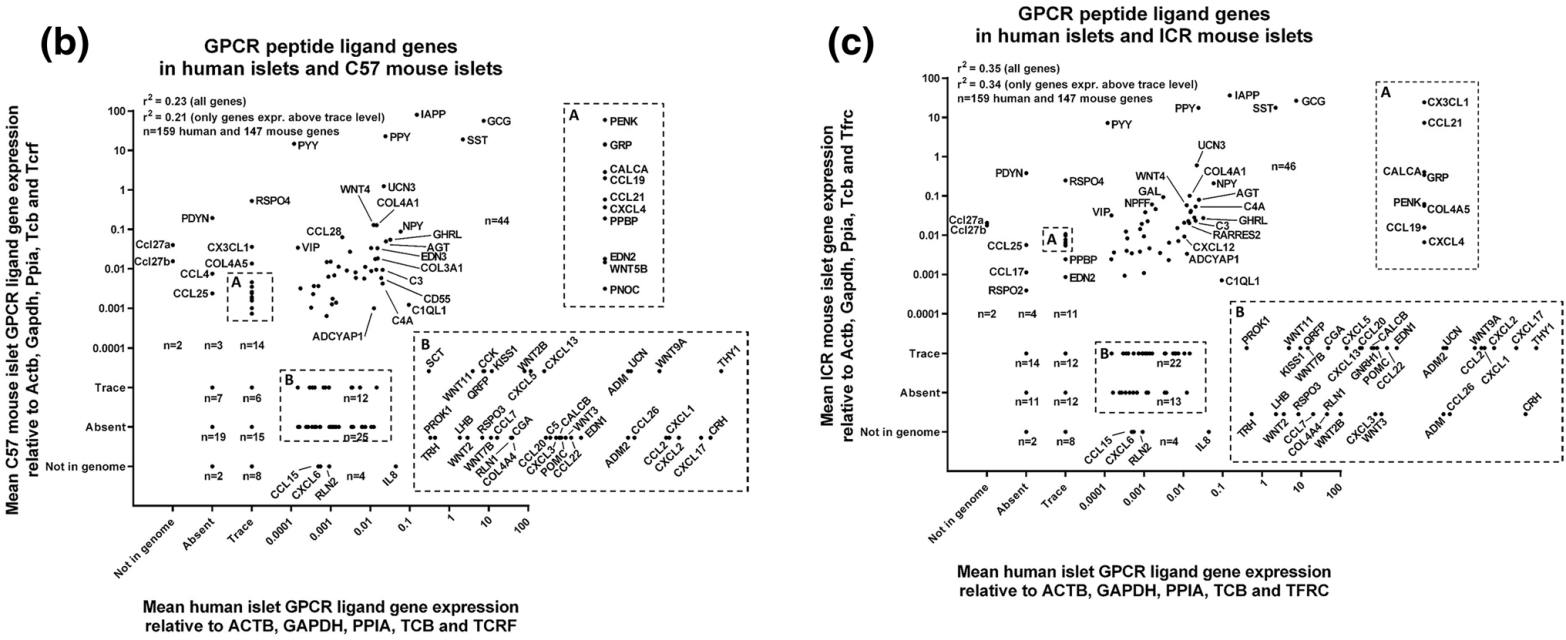
Mean relative mRNA expression of GPCR peptide ligand genes in human and ICR and C57 mouse islets relative to the reference genes Actb, Gapdh, Ppia, Tbp and Tfrc. Insert panels A and B in **b**, **c** represent enlarged areas of the scatter plots, added to allow visualisation of individual mRNAs. **a** Data for 147 GPCR peptide ligand genes from non-pooled biological replicates from islets isolated from four C57 and four ICR mice. **b** Data for 159 human and 147 mouse GPCR peptide ligand genes from non-pooled biological replicates from four C57 and four human islet preparations. **c** Data for 159 human and 147 mouse GPCR peptide ligand genes from non-pooled biological replicates from four ICR and four human islet preparations

Of the 159 human GPCR peptide ligand genes screened, mRNAs encoding 128 were detected in human islets and, of the 147 mouse GPCR peptide ligands, mRNAs encoding 111 and 88 were detected in outbred ICR mouse islets and inbred C57 mouse islets, respectively. As expected, genes encoding glucagon and somatostatin were two of the most abundant GPCR peptide ligands identified in human and mouse islets, and mRNA for islet amyloid polypeptide (IAPP) was highly expressed in mouse islets, with lower levels detected in human islets (Fig. 1). Two additional GPCR peptide ligand genes (PPY and UCN3) were among the top ten in both human and mouse islets, indicative of close correlation between species in islet expression of abundant secretory peptide mRNAs (Fig. 1). Nonetheless, Pyy (peptide YY), which was the fifth most abundant GPCR peptide ligand gene in both C57 and ICR mouse islets, was not highly expressed by human islets. In addition, the intra-species secretome profiles (*r*^2^ = 0.97) were of greater similarity than those between species, with islets from the two mouse strains sharing nine out of ten of the most abundantly expressed GPCR peptide ligand genes (Col4a1, Gcg, Iapp, Pdyn, Ppy, Pyy, Rspo4, Sst, Ucn3). Only five genes were preferentially expressed in either of the two mouse strains: Cck, Ccl17, Hc, Rspo2 and Sct mRNAs were identified above trace level only in ICR mouse islets, while Ccl4, Gnrh1, Insl5, Pnoc and Wnt5b mRNAs were only quantifiable in C57 mouse islets (Fig. 2a). As demonstrated in Fig. 2b, c, comparison of islet mRNA expression of all GPCR peptide ligands between human islets and the two mouse strains indicated lower correlation than for the inter-strain comparison (inbred C57 mouse islets: *r*^2^ = 0.23; outbred ICR mouse islets: *r*^2^ = 0.35).

**Fig. 3:**
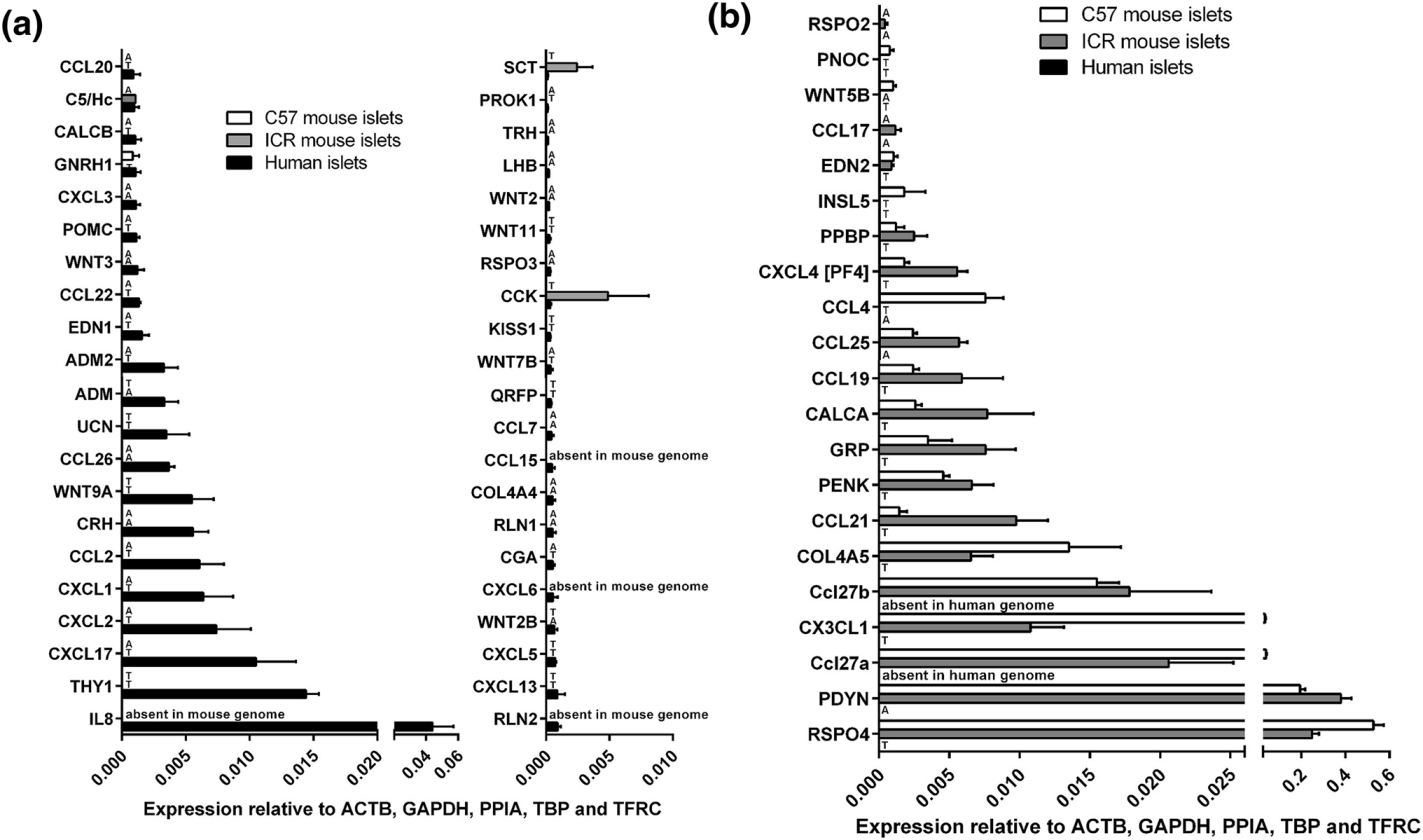
Species- and strain-specific enrichment of GPCR peptide ligand genes. Data are presented as mean + SEM relative to Actb, Gapdh, Ppia, Tbp and Tfrc for four human islet donors and four ICR and four C57 mouse islet preparations. T denotes trace expression and A denotes absent expression. **a** Expression of 42 GPCR peptide ligand genes that are expressed above trace level in human islets, but either absent or present only at trace level in either ICR or C57 mouse islets. **b** Expression of 21 GPCR peptide ligand genes that are expressed above trace level in ICR or C57 mouse islets, but either absent or present only at trace level in human islets

Deeper analysis of all data indicated that over 40 of the mRNAs encoding human GPCR peptide ligands were also expressed above trace level in mouse islets (C57: 44 genes; ICR: 46 genes), while 42 mRNAs were quantifiable in human islets but absent or at trace levels in the mouse strains. In particular, thy-1 membrane glycoprotein (THY1) and the chemokines CXCL8 (IL-8), CXCL17, CXCL2, CXCL1 and CCL2 were the most abundantly expressed human-specific GPCR peptide ligands, as shown in Fig. 3a. Conversely, mRNAs encoding 21 GPCR peptide ligand genes were either absent or unquantifiable in human islets but expressed above trace level in at least one of the two mouse strains, with R-spondin-4 (Rspo4), prodynorphin (Pdyn) and the chemokines Ccl27a, Cx3cl1 and Ccl27b being the most abundant mouse islet-specific GPCR ligand mRNAs (Fig. 3b).

**Fig. 4:**
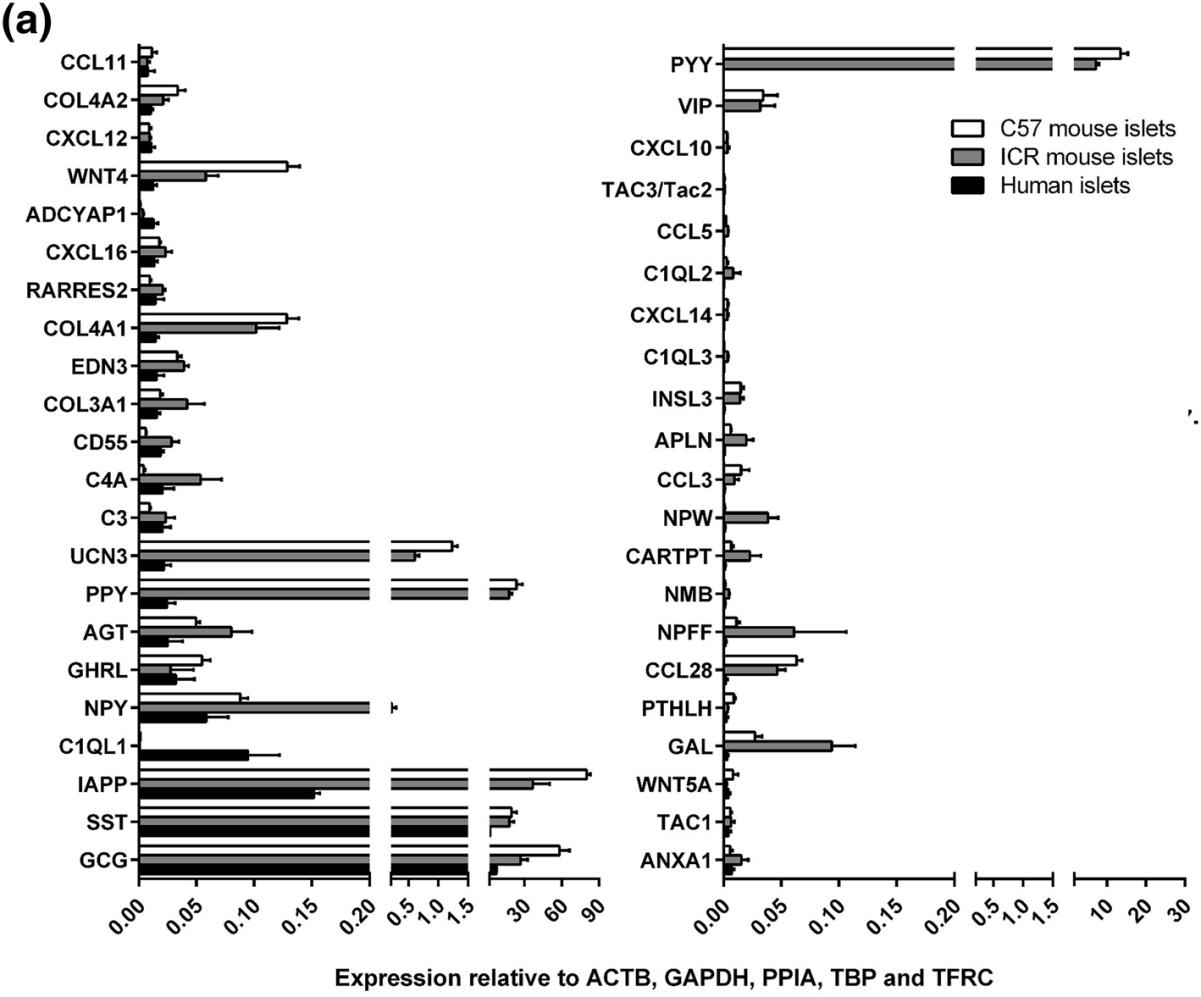

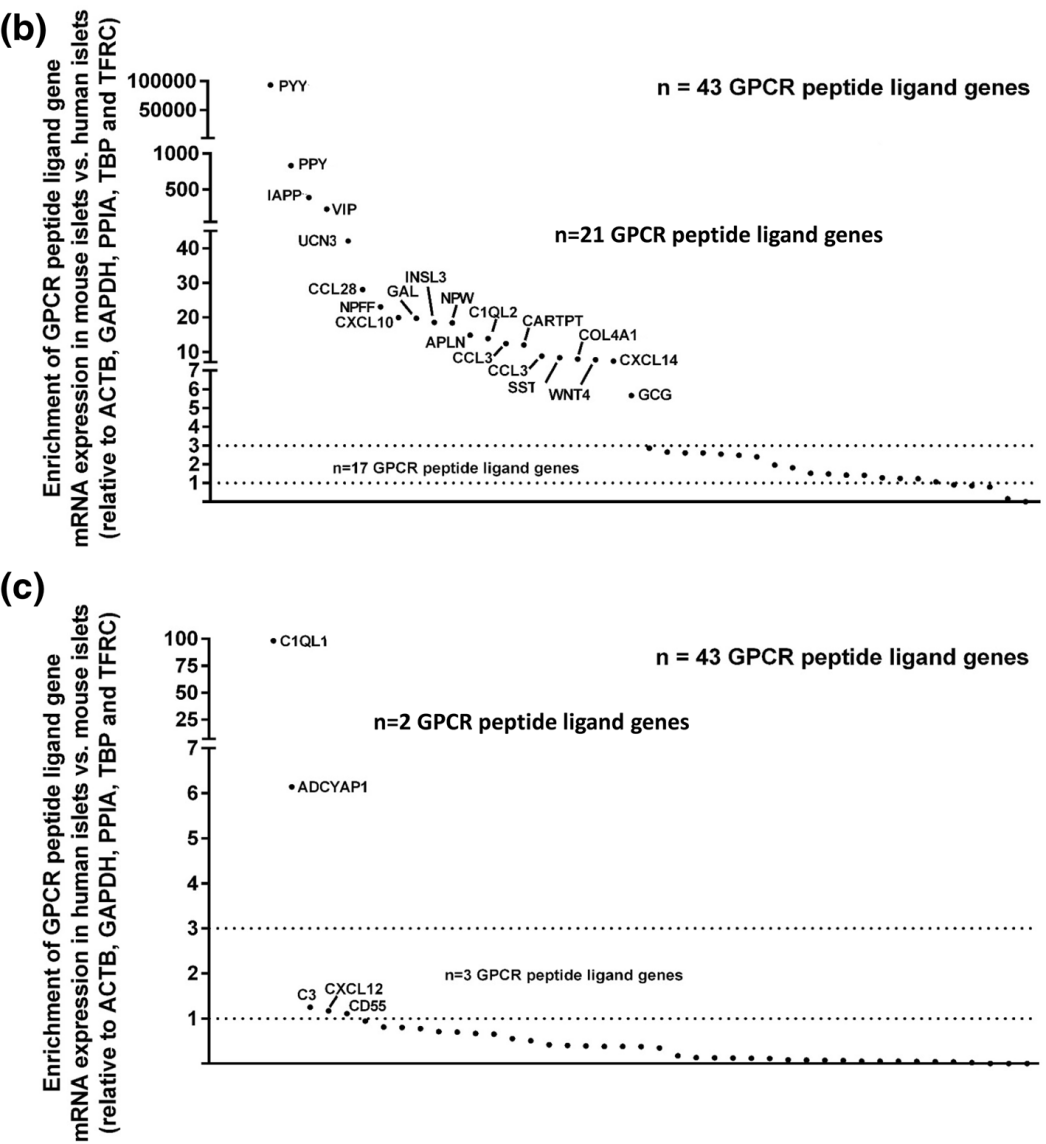
Peptide ligands expressed above trace level in human and mouse islets. Data in **a** are presented as mean + SEM relative to Actb, Gapdh, Ppia, Tbp and Tfrc and data in **b**, **c** are ratios of the mean expression relative to Actb, Gapdh, Ppia, Tbp and Tfrc. Data were generated using non-pooled islet preparations from four ICR mice, four C57 mice and four human donors. **a** Expression of 43 GPCR ligand genes that are expressed above trace level in mouse (ICR and C57) and human islets. **b** Species enrichment of GPCR peptide ligand genes present above trace level in mouse islets. **c** Species enrichment of GPCR peptide ligand genes present above trace level in human islets

The 43 GPCR peptide ligand mRNAs that were expressed above trace level in both human and mouse islets are shown in Fig. 4a. Of these, 21 mRNAs were > threefold enriched in mouse islets, with peptide YY (PYY) showing ~ 40,000-fold higher expression in mouse islets than in human islets, followed by pancreatic polypeptide (PPY), islet amyloid polypeptide (IAPP) and vasoactive intestinal peptide (VIP) (Fig. 4b). In human islets, mRNAs encoding complement component 1, q subcomponent-like 1 (C1QL1) and adenylate cyclase activating polypeptide 1 (ADCYAP1) were enriched by ~ 100-fold and 6-fold compared to mouse islets, while all other GPCR ligand mRNAs were expressed at levels similar to, or lower than, those observed in mouse islets (Fig. 4c). Supplementary Fig. 1 shows relative expression of all of the GPCR peptide ligand genes that were quantified in human and mouse islets, placed alphabetically into their peptide sub-families, to provide a readily assimilated overview of expression patterns.

### Comparative analysis of GPCR/peptide signalling pathways in human and mouse islets

**Fig. 5:**
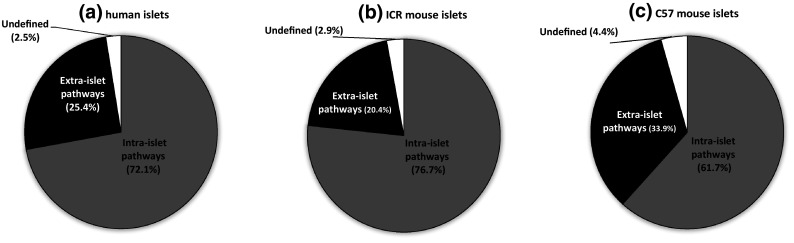
Summary of intra- and extra-islet peptide/GPCR signalling pathways in human islets (**a**) and in ICR (**b**) and C57 (**c**) mouse islets. Data are presented as % of the pathways that occur within islets (intra-islet), where the ligands are released from islets to act at non-islet GPCRs (extra-islet), or where the peptide ligands have been proposed to interact with as yet unidentified GPCRs (undefined)

The quantification of mouse and human islet GPCR peptide ligand mRNAs, together with our earlier quantification of islet GPCR mRNA expression [1, 11], allowed us to generate signalome atlases that defined 418 peptide ligand/GPCR signalling pathways in human and mouse islets (Supplementary Fig. 2). This figure illustrates in detail the pathways through which families of peptides that are synthesised and secreted from islets may interact in an autocrine or paracrine manner with local islet GPCRs, and it also indicates that the peptides may have distal effects through activation of non-islet GPCRs. Our data indicate substantial local “intra-islet” signalling pathways, with mRNAs encoding both the peptide ligand and its cognate receptor being expressed by islets (human islets: 256 pathways; ICR mouse islets: 240 pathways; C57 mouse islets: 140 pathways; Fig. 5a–c, Supplementary Fig. 2). “Extra-islet” signalling, which is defined as pathways where islets do not express the cognate GPCRs for peptide ligands synthesised by islets, was less common than intra-islet signalling, with approximately 1/5–1/3 of pathways in this category (Fig. 5).

Comparison of relative expression of GPCR peptide ligand and GPCR mRNAs in the signalome atlases led to the identification of species differences in mRNA expression, which can be used to predict the relative importance of particular pathways. For example, mRNAs encoding peptide YY (Pyy) and pancreatic polypeptide (Ppy), which are ligands at NPY receptors, were expressed at ~ 40,000-fold (Pyy) and ~ 400-fold higher levels in mouse islets than in human islets (Figs. 3, 4, Supplementary Figs. 1, 2). In addition, the prodynorphin (Pdyn) gene, which encodes several peptides of the dynorphin family that act as ligands at δ, μ and κ opioid receptors, was abundantly expressed in mouse islets but absent in human islets. Similarly, mRNA encoding the small secreted protein R-spondin, which acts as an agonist of the leucine-rich repeat GPCRs Lgr4, Lgr5 and Lgr6, was present at high levels in mouse islets but only found at trace levels in human islets. In contrast, our analysis indicated that human islets express high levels of mRNA encoding C1QL1, while mouse islets express very low levels of this mRNA. A similar pattern was observed for the CXCL8 gene that encodes the chemotactic peptide IL-8: CXCL8 is absent from the mouse genome and, therefore, was not present in mouse islet cells, but CXCL8 mRNA was abundantly expressed by human islets. As human islets do not express mRNAs encoding the CXCR1 and CXCR2 receptors, through which IL-8 signals, it is likely that this peptide may have an extra-islet role, perhaps in local inflammation, following its secretion from human islet cells. Overall, the signalomes depicted in Supplementary Fig. 2 allow us to predict that the C1QL1 signalling pathway may dominate in human islets, while the PYY, pancreatic polypeptide, dynorphin and R-spondin 4 signalling pathways are more important in regulating function of mouse islets.

### PYY as a comparative example for ligand peptide GPCR expression in human and mouse islets

**Fig. 6:**
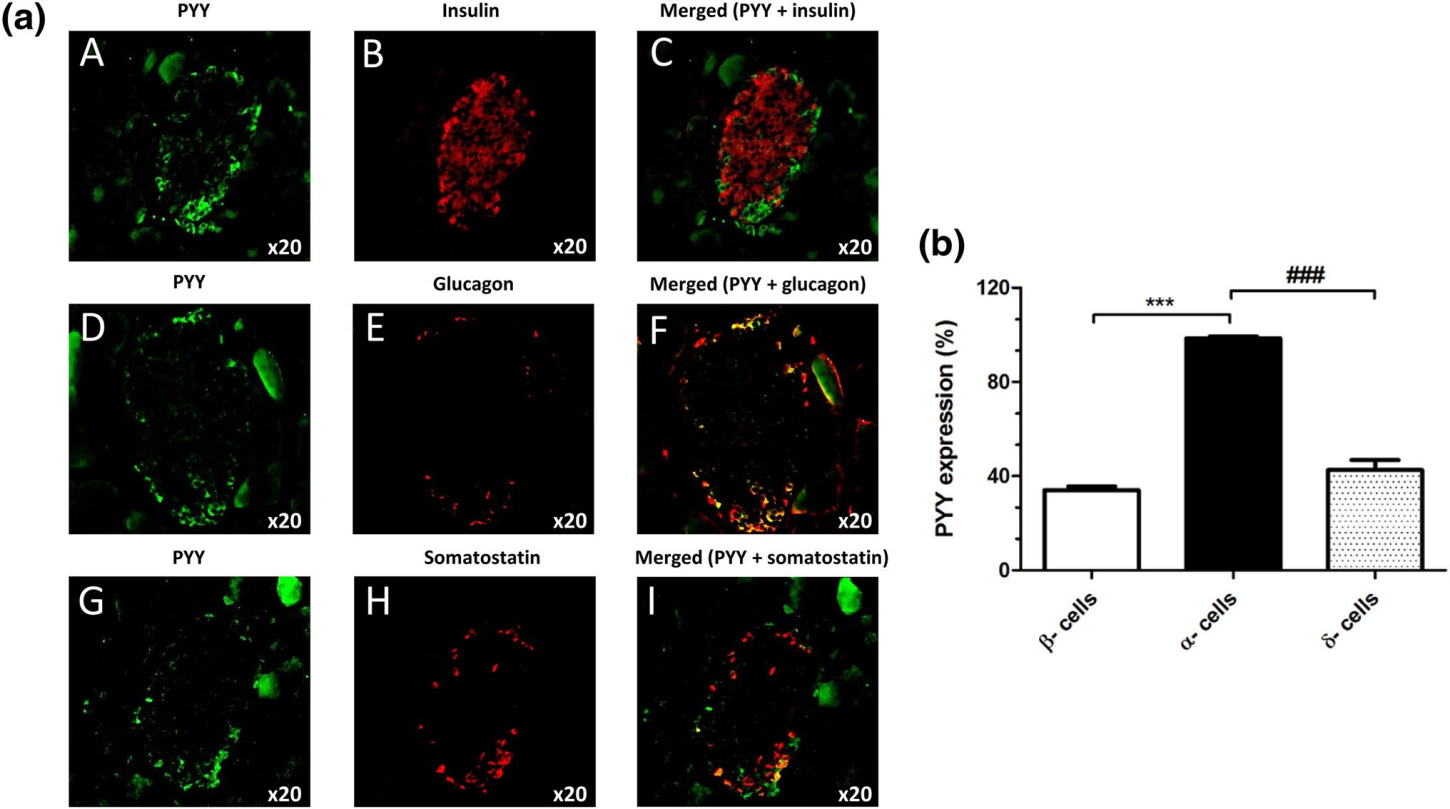

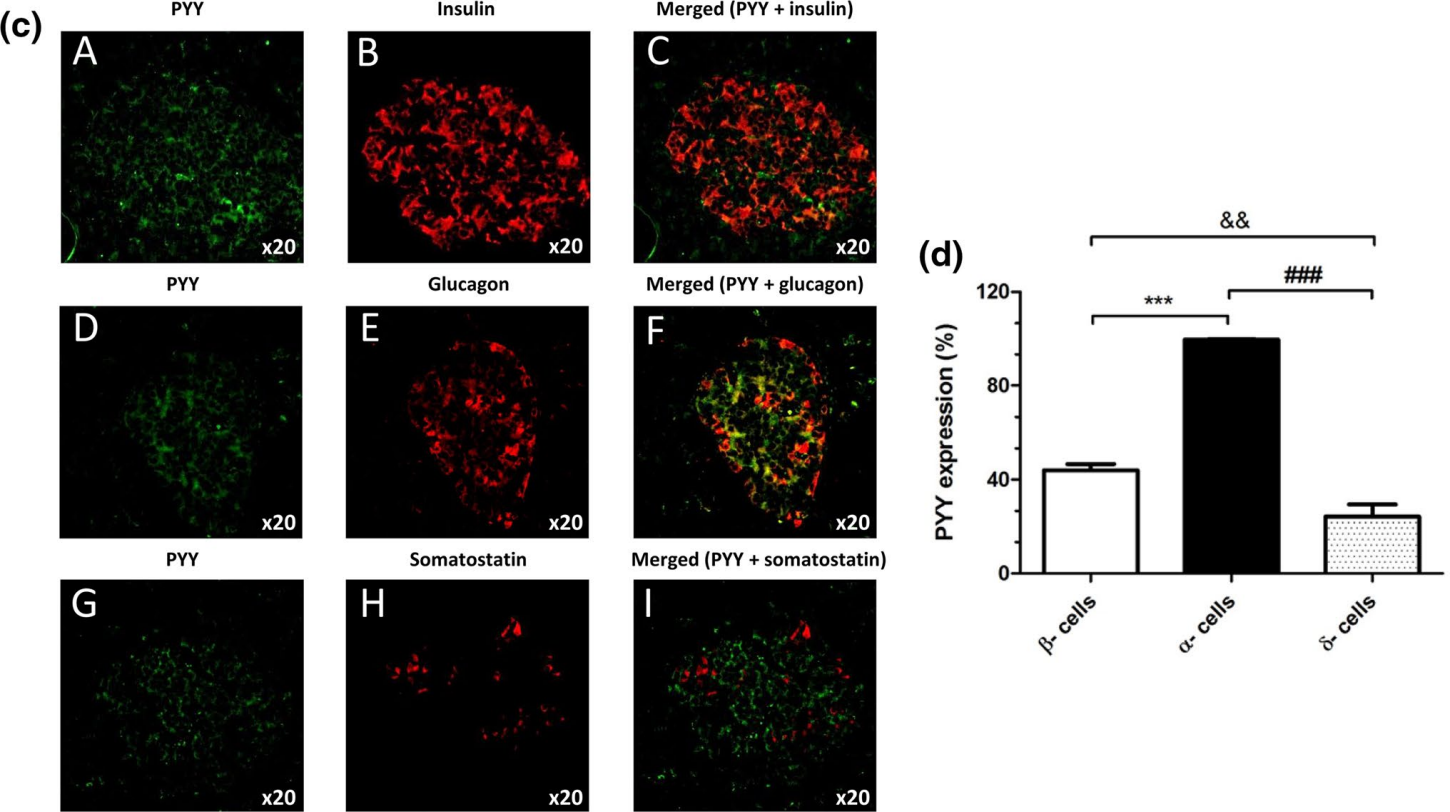
Expression of PYY protein in mouse and human pancreas sections. Co-localisation of PYY (green) with the islet hormones insulin (**A**–**C**), glucagon (**D**–**F**) and somatostatin (**G**–**I**) (red) in mouse (**a**) and human (**c**) islets. The data in **b**, **d** indicate quantification of the percentage of mouse (**b**) and human (**d**) islet endocrine cells that express PYY. ^&&^*p* < 0.01, ***^,###^*p* < 0.001. *n* = 7–9 islets

Fluorescence immunohistochemical staining of mouse and human pancreas sections indicated that PYY protein was present in mouse (Fig. 6a, b) and human (Fig. 6c, d) islets, with more intense immunostaining in mouse islets. Co-staining with antibodies against insulin, glucagon and somatostatin demonstrated that in both species PYY co-localised primarily with α-cells and low expression of this protein was also detected in β- and δ-cells (Fig. 6b, d).

**Fig. 7:**
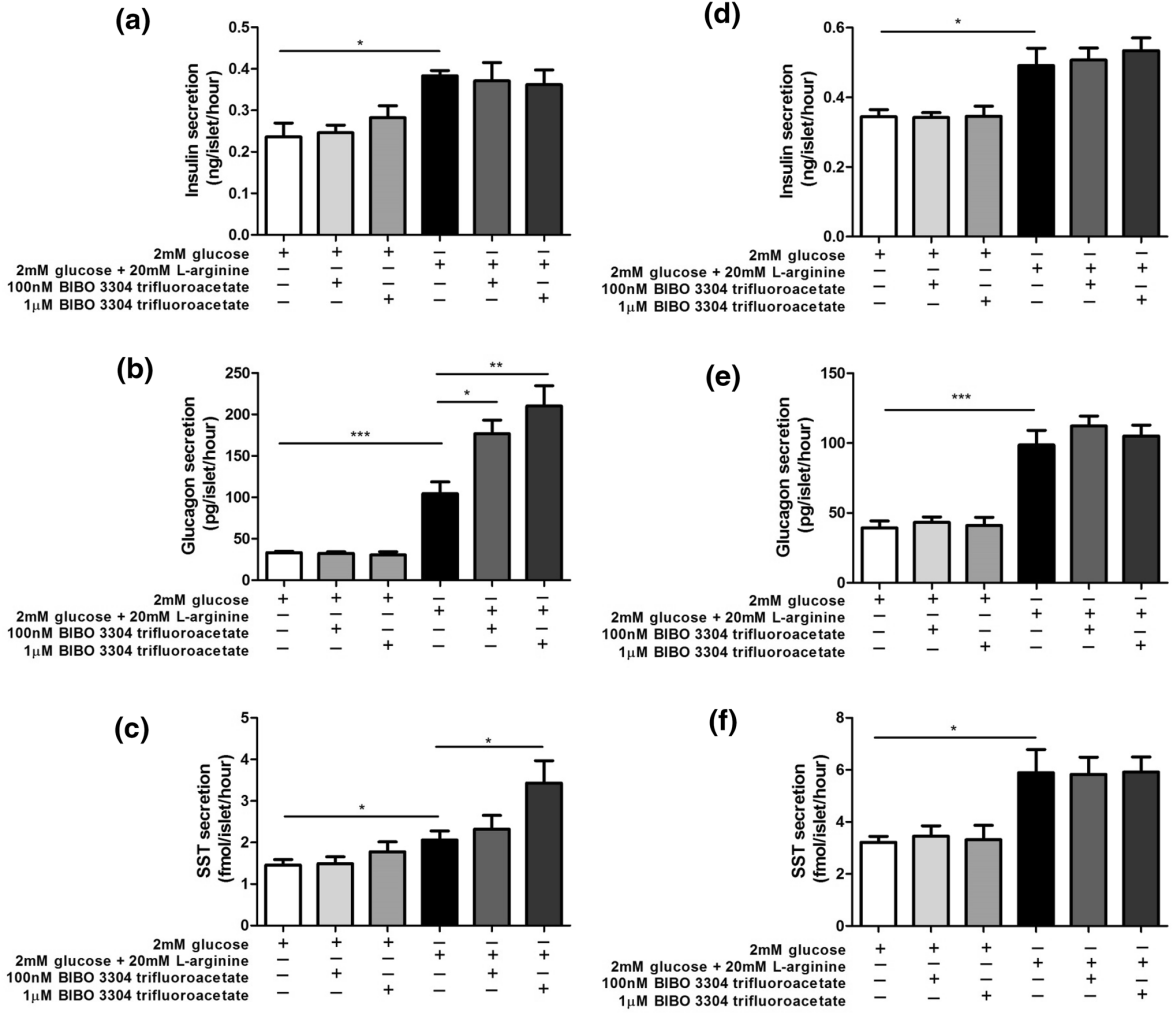
Effect of BIBO 3304 trifluoroacetate on insulin (**a**, **d**), glucagon (**b**, **e**) and somatostatin (**c**, **f**) secretion from mouse (**a**–**c**) and human (**d**–**f**) islets. Data are shown as mean + SEM insulin, glucagon and somatostatin release per islet per hour in static incubations, *n* = 8 for all hormones in both mouse and human islets. *p < 0.05, **p < 0.01; ***p < 0.001

NPY1R is the major NPY receptor expressed by mouse and human islets [11, 26] and activation of this Gi-coupled receptor is reported to inhibit insulin secretion [27]. BIBO 3304 trifluoroacetate (BIBO), a NPY1R antagonist, was therefore used to block the effects of PYY released from islet cells in response to 20 mM l-arginine, and hormone secretion from isolated mouse and human islets was quantified by radioimmunoassay. l-Arginine-stimulated insulin secretion from mouse and human islets, and BIBO 3304 trifluoroacetate had no effect on this secretory response when used at concentrations of 100 nM and 1μM (Fig. 7a, d). However, BIBO 3304 trifluoroacetate significantly potentiated l-arginine-induced glucagon and somatostatin secretion from mouse islets (Fig. 7b, c), but was without effect on secretion of these hormones from human islets (Fig. 7e, f).

## Discussion

Peptides and small proteins are important regulators of islet function and glucose homeostasis, at least in part through interactions with GPCRs present both on islet cells and other organ systems [1]. However, although human islets express 293 GPCRs, to date the GLP-1 receptor is the only islet GPCR that is currently a target for drugs used to treat type 2 diabetes [28, 29]. In addition to expression of numerous GPCRs, islets are thought to express mRNAs of > 100 genes encoding peptide and protein ligands of GPCRs [1], some of which may have potential as novel diabetes therapies. In the current study, we used qPCR with validated primers for GPCR peptide ligand mRNAs as a versatile yet cost effective and sensitive method for detection of low-abundant transcripts that are not easily detectable using low throughput antibody-based approaches [19]. The data generated were used to construct three islet signalome atlases describing all known GPCR/peptide signalling pathways in human islets and islets from outbred ICR and inbred C57 mice, to allow identification of similarities and differences in GPCR peptide ligand expression between the two species and place these differences into a pharmacological perspective.

In total, the signalome atlases indicated the existence of 418 signalling pathways through which human- and mouse islet-derived peptides could interact with GPCRs, approximately 20% of which were conserved between species. An additional 50% of all identified pathways shared a large number of components between human islets and islets from the two mouse strains, but were not identical. About 4% of all GPCR/peptide signalling pathways were only found in mouse islets, whereas 20% were unique to human islets. The majority of the pathways identified constitute endogenous islet signalling in which the mRNAs encoding both the peptide ligand and the corresponding cognate GPCR were expressed by islet cells. Although many of these GPCRs are also expressed in other cell types and organ systems, it is likely that the islet-derived peptides will have local effects on the islet GPCRs before being transported by the systemic circulation to other cells expressing the receptors.

When considering genes encoding specific GPCR peptide ligands, it was found that glucagon and islet amyloid polypeptide (Iapp) mRNAs showed the highest expression in human and mouse islets, respectively. These observations are consistent with human islets having a greater proportion of α-cells than mouse islets [13, 14] and with Iapp being synthesised and co-stored with insulin in β-cells [30], which constitute approximately 80% of the endocrine cells of mouse islets [13, 14]. While mouse and human islets express very high levels of preproinsulin mRNA this was not quantified in the current study as insulin is an agonist at a tyrosine kinase receptor rather than a GPCR. The peptide ligand mRNA expression profile of ICR mouse islets was closer than that of C57 mouse islets to the mRNA expression of human islets, most likely as a consequence of normal genetic variation being retained better in outbred ICR mice than in the more homogenous inbred C57 mice. Interestingly, we found that several genes encoding ‘classical’ islet peptides, such as peptide YY (PPY), pancreatic polypeptide (PPY), vasoactive intestinal polypeptide (VIP) and urocortin 3 (UCN3) showed considerably higher expression in mouse than human islets. In particular, the gene encoding PYY was ~ 40,000-fold more abundant in mouse islets. Similarly, mRNAs encoding R-spondin 4 (RSPO4) and dynorphin peptides (PDYN) were expressed at very high levels in mouse islets, but were either absent or present only at trace levels in human islets. In contrast, mRNAs encoding the small secreted protein C1QL1 and the chemokine IL-8 were enriched in human islets.

The observation that PYY mRNA expression was considerably higher in mouse islets than in human islets led us to determine whether this 36-amino acid peptide that binds to the neuropeptide Y family of GPCRs (NPY1R, NPY2R, NPY4R, NPY5R) to regulate food intake and energy homeostasis [31] has differential functional effects in mouse and human islets. Fluorescence immunohistochemistry indicated higher PYY immunoreactivity in mouse than human islets, and co-staining with antibodies against islet hormones demonstrated that it was mainly localised to mouse and human islet α-cells, with lesser expression in β- and δ-cells. The main source of PYY is from enteroendocrine L-cells in the gastrointestinal tract following food intake, and it is rapidly cleaved N-terminally such that the major circulating form is PYY_3–36_ [31, 32]. This truncated peptide preferentially activates NPY2R, which is absent from mouse and human islets [1, 11, 33], so it is likely that islet-derived, full-length PYY (PYY_1–36_), which has high affinity for NPY1R, may exert local effects following its release from mouse islets.

We used l-arginine, an amino acid that induces islet endocrine cell exocytosis, to maximise PYY release from islets then blocked action of locally secreted PYY with the selective NPY1R antagonist BIBO. PYY_1–36_ is reported to inhibit insulin secretion through NPY1R [31, 32, 34–37] so it was expected that exposure of arginine-stimulated mouse islets to BIBO would have enhanced insulin release, as has been observed in experiments where this antagonist blocked the inhibitory effects of exogenous PYY [37]. However, neither 100 nM nor 1μM BIBO significantly affected insulin secretion, suggesting that local PYY concentrations were not sufficiently high to reduce arginine-induced insulin release. In contrast, we found that BIBO caused a concentration-dependent potentiation of arginine-stimulated glucagon release from mouse islets, consistent with the reported effect of PYY to inhibit glucagon secretion [34]. As expected, given the lower levels of PYY in human islets, BIBO did not increase glucagon release in the human islet experiments. There have been no previous reports on the effects of PYY on somatostatin secretion, but our data suggest that locally released PYY acts at δ-cell NPY1R to inhibit somatostatin release, which is antagonised by BIBO. Again, this effect was not observed in human islets, which contain less PYY. Overall, these data point to a role for intra-islet signalling by PYY in mouse but not human islets.

In summary, in this study we have systematically compared the expression of all known human GPCR peptide ligand mRNAs with their mouse orthologues in human and mouse islets, and created detailed islet GPCR/peptide ligand signalome atlases to illustrate the similarities and differences in GPCR peptide ligand expression and signalling pathways. We have used the signalomes to predict a more important role for islet-derived PYY in mouse than human islets, and confirmed this by functional studies with an NPY1R antagonist. The data presented here will allow researchers to focus on GPCR peptide ligands that are present in human islets but not in mouse, perhaps leading to development of novel diabetes therapeutics.

### Electronic supplementary material

Below is the link to the electronic supplementary material.

Supplementary material 1 (PPTX 2145 kb) **Supplementary Fig.** **1.** Expression of human GPCR peptide ligand genes and their mouse orthologues in human and mouse islets. Data are relative to Actb, Gapdh, Ppia, Tbp and Tfrc, and were generated using non-pooled islet preparations from four ICR mice, four C57 mice and four human donors. T: trace mRNA expression; A: mRNA absent

Supplementary material 2 (PPTX 247 kb) **Supplementary Fig.** **2.** Human and mouse islet signalome atlases outlining 418 peptide ligand/GPCR signalling pathways in human and mouse islets. Separate pathways were constructed based on quantification of mRNAs encoding the peptide ligands and their receptors in human and mouse (ICR and C57) islets. Dominant pathways, based on high expression levels, are indicated by bold arrows and text. Grey boxes: GPCR not expressed in islets. Grey text: GPCR or peptide ligand mRNA present only at trace levels. *Several isoforms exist of the mature peptide
