# Supplementary figures and images for: Defining G protein-coupled receptor peptide ligand expressomes and signalomes in human and mouse islets
Source: Cell Mol Life Sci. 2018 Feb 17;75(16):3039–50. doi: 10.1007/s00018-018-2778-z (PMC6061145; doi:10.1007/s00018-018-2778-z)

## Slide 1
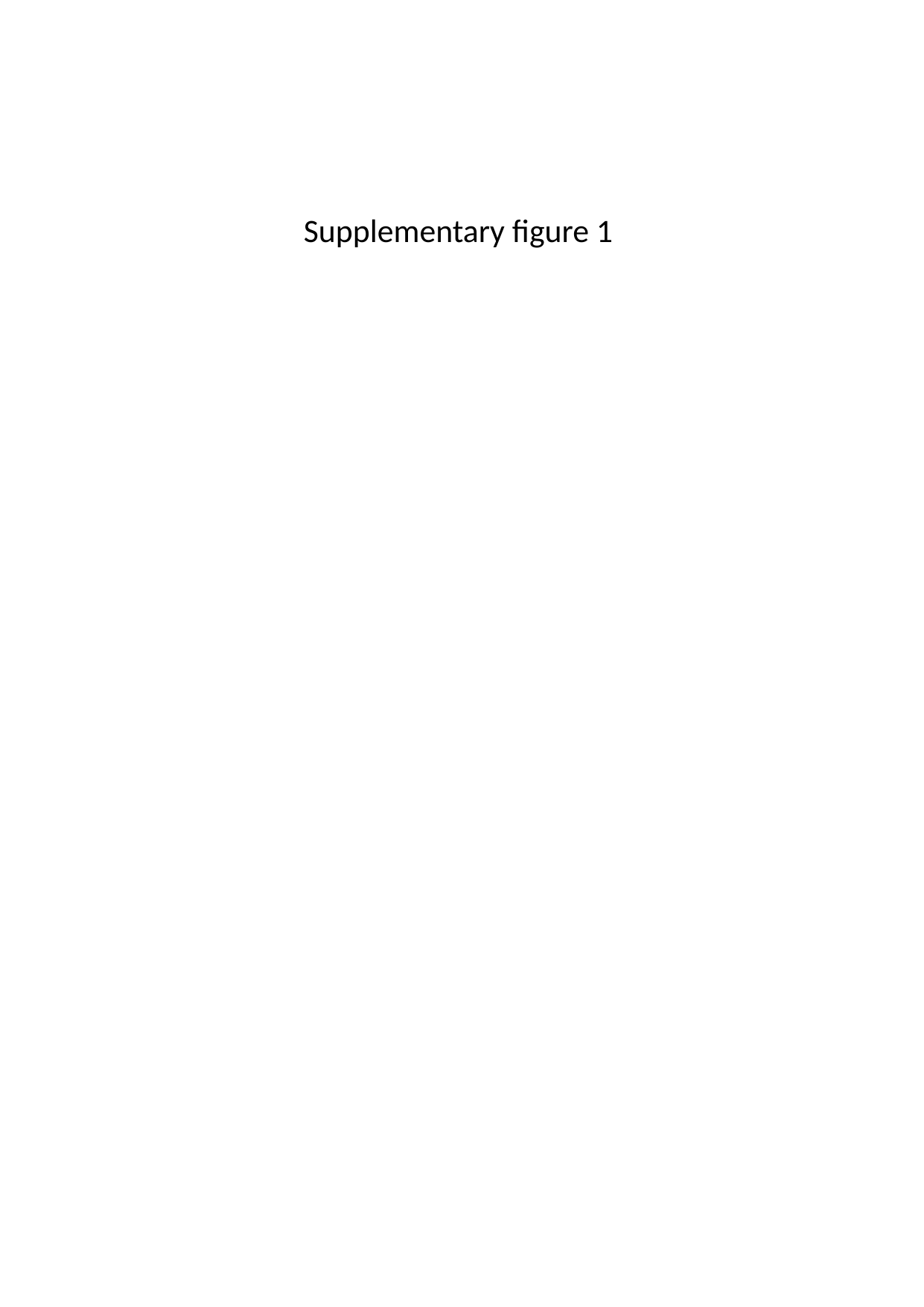

Supplementary figure 1

## Slide 2
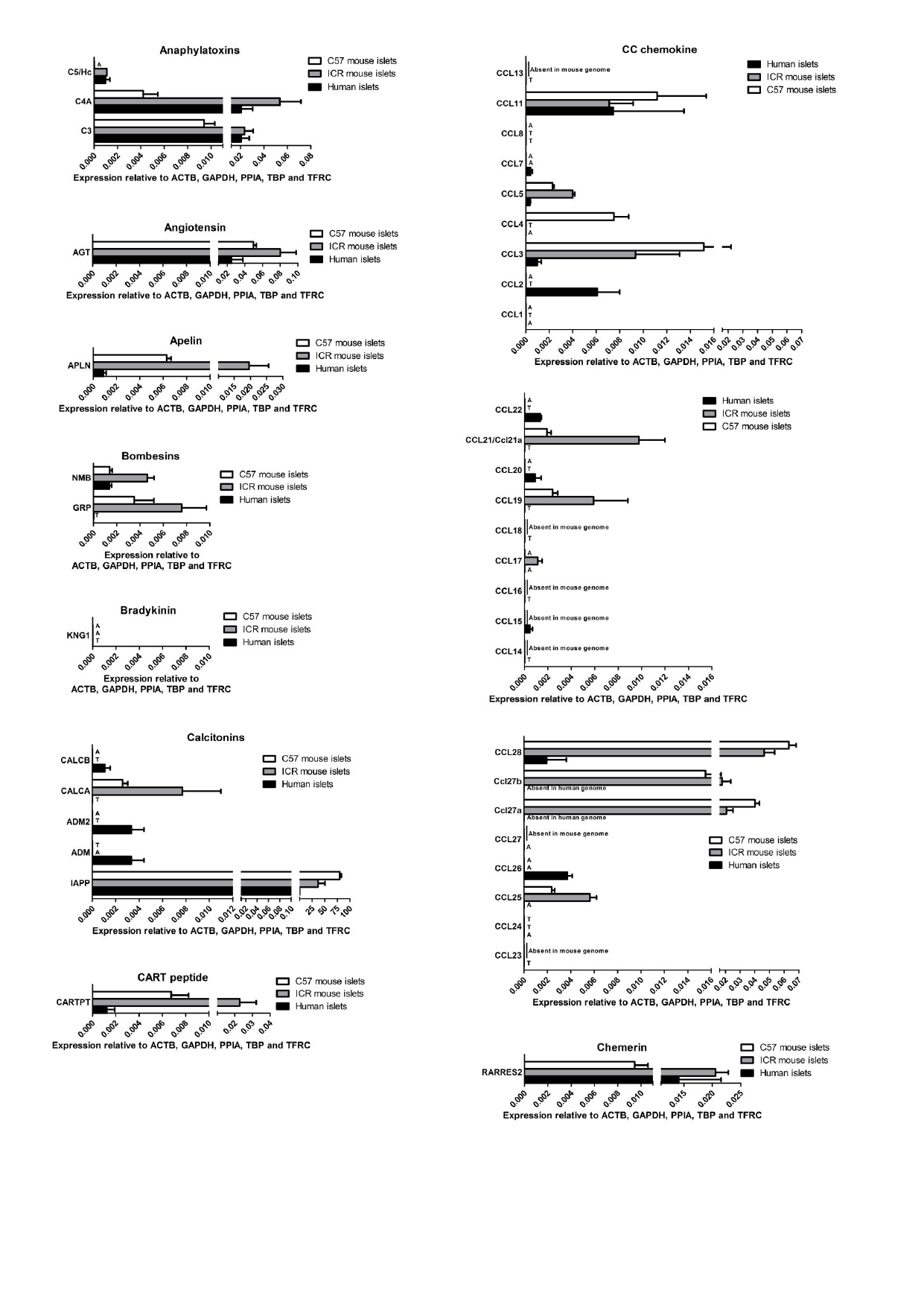

## Slide 3
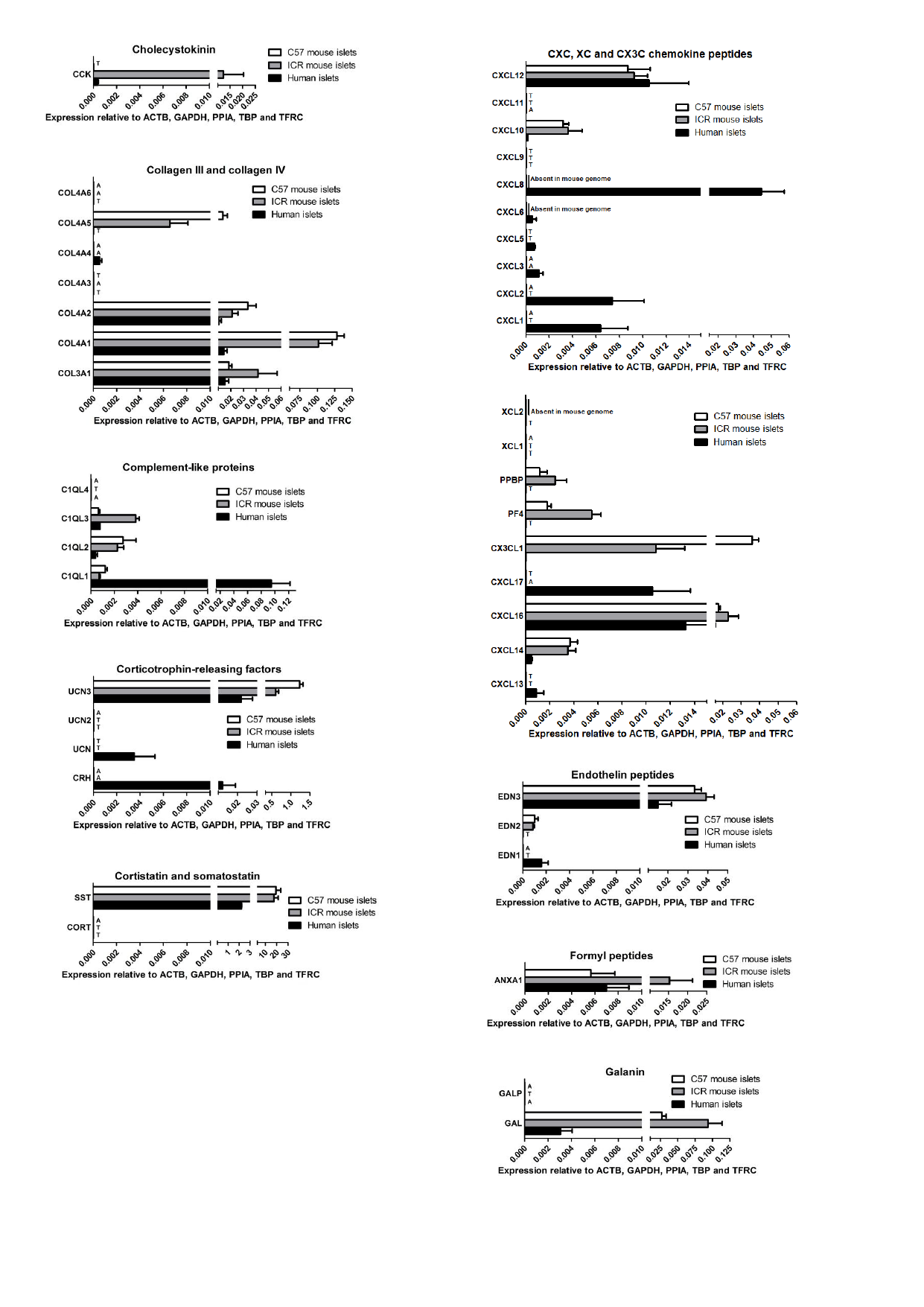

## Slide 4
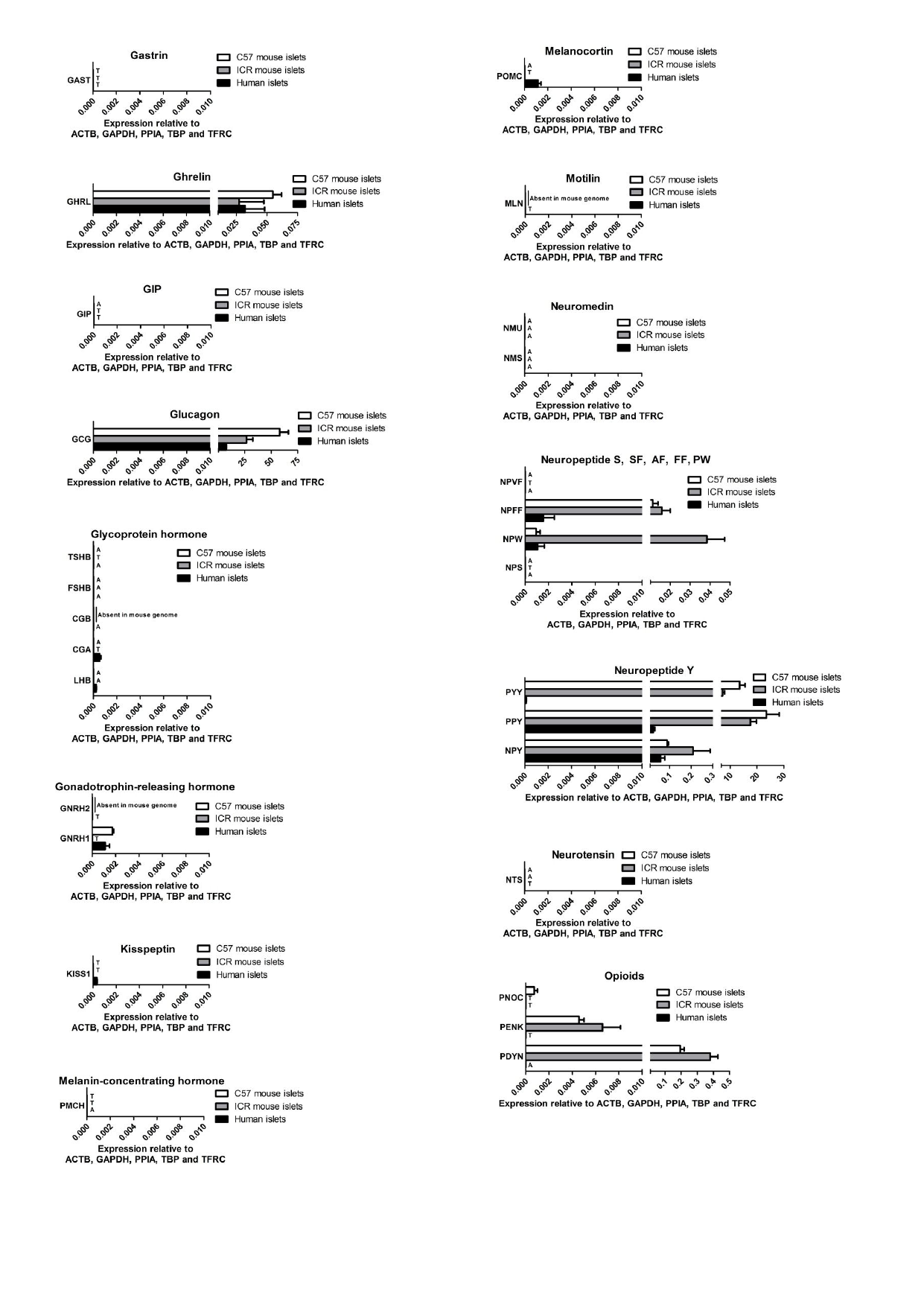

## Slide 5
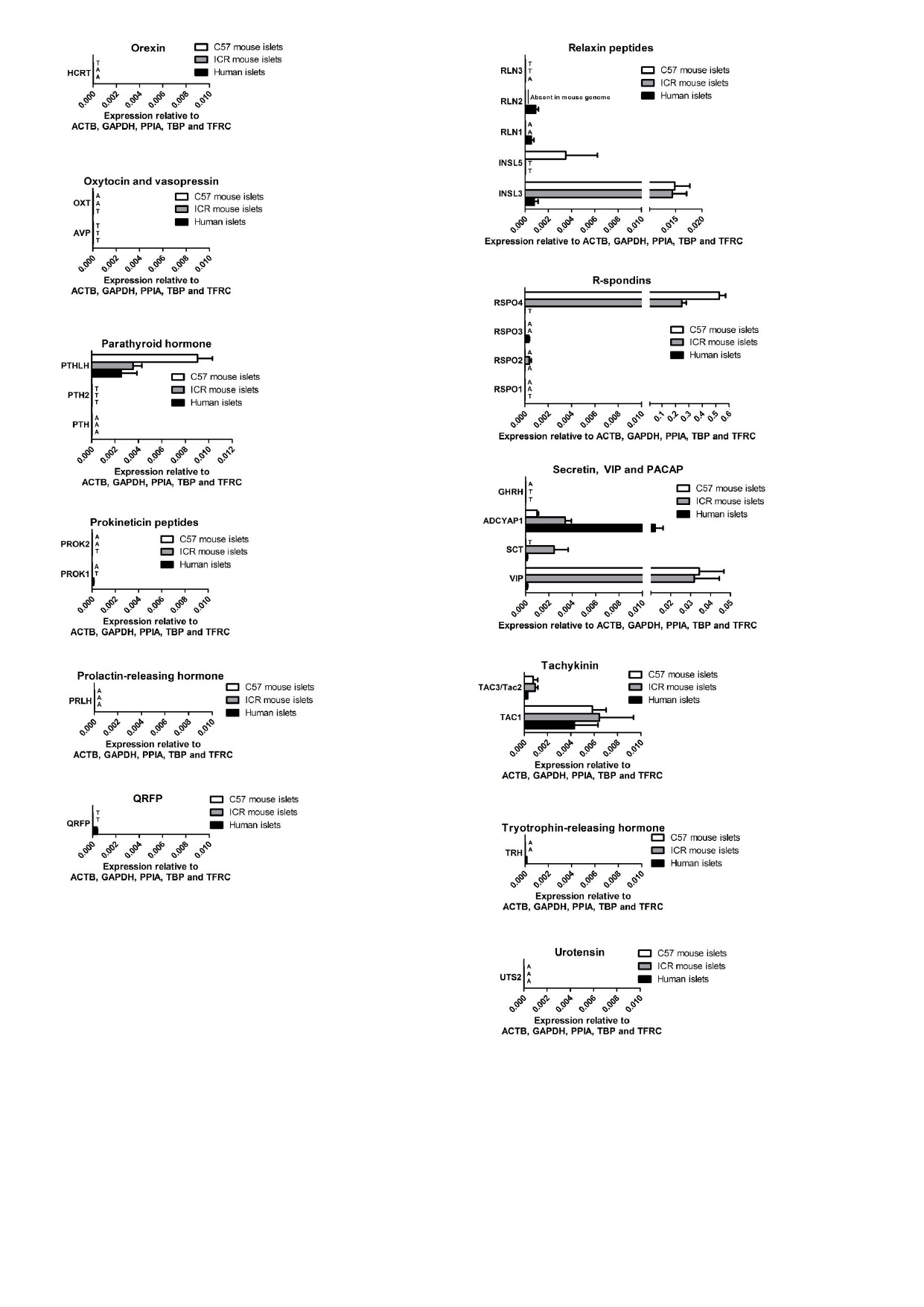

## Slide 6
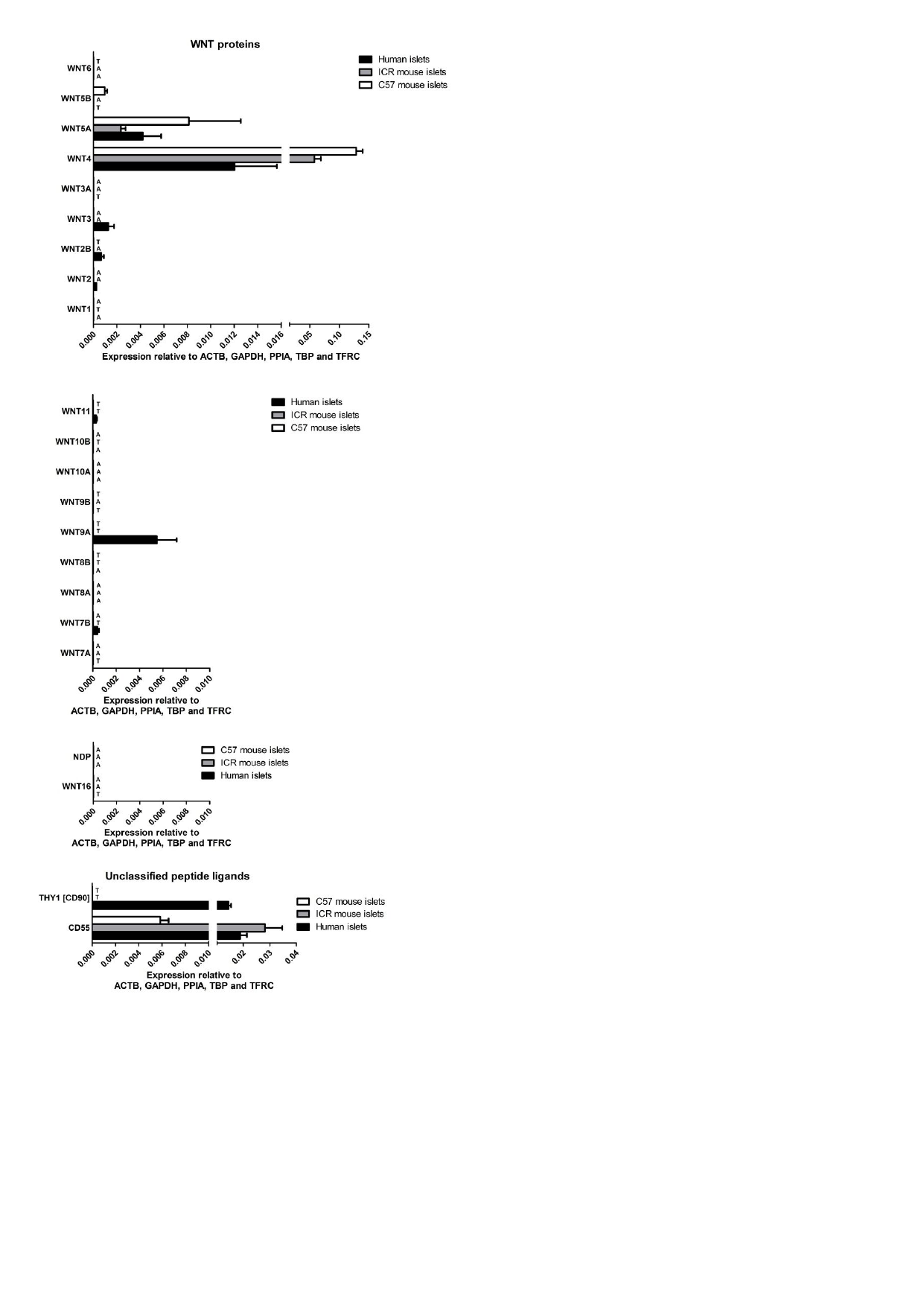

Supplement: Supplementary file 1 — Supplementary material 1 (PPTX 2145 kb) Supplementary Fig. 1. Expression of human GPCR peptide ligand genes and their mouse orthologues in human and mouse islets. Data are relative to Actb, Gapdh, Ppia, Tbp and Tfrc, and were generated using non-pooled islet preparations from four ICR mice, four C57 mice and four human donors. T: trace mRNA expression; A: mRNA absent [file 18_2018_2778_MOESM1_ESM.pptx]
