# Supplementary material for: Defining G protein-coupled receptor peptide ligand expressomes and signalomes in human and mouse islets
Source: Cell Mol Life Sci. 2018 Feb 17;75(16):3039–50. doi: 10.1007/s00018-018-2778-z (PMC6061145; doi:10.1007/s00018-018-2778-z)
Supplement: Supplementary file 2 — Supplementary material 2 (PPTX 247 kb) Supplementary Fig. 2. Human and mouse islet signalome atlases outlining 418 peptide ligand/GPCR signalling pathways in human and mouse islets. Separate pathways were constructed based on quantification of mRNAs encoding the peptide ligands and their receptors in human and mouse (ICR and C57) islets. Dominant pathways, based on high expression levels, are indicated by bold arrows and text. Grey boxes: GPCR not expressed in islets. Grey text: GPCR or peptide ligand mRNA present only at trace levels. *Several isoforms exist of the mature peptide [file 18_2018_2778_MOESM2_ESM.pptx]

## Slide 1
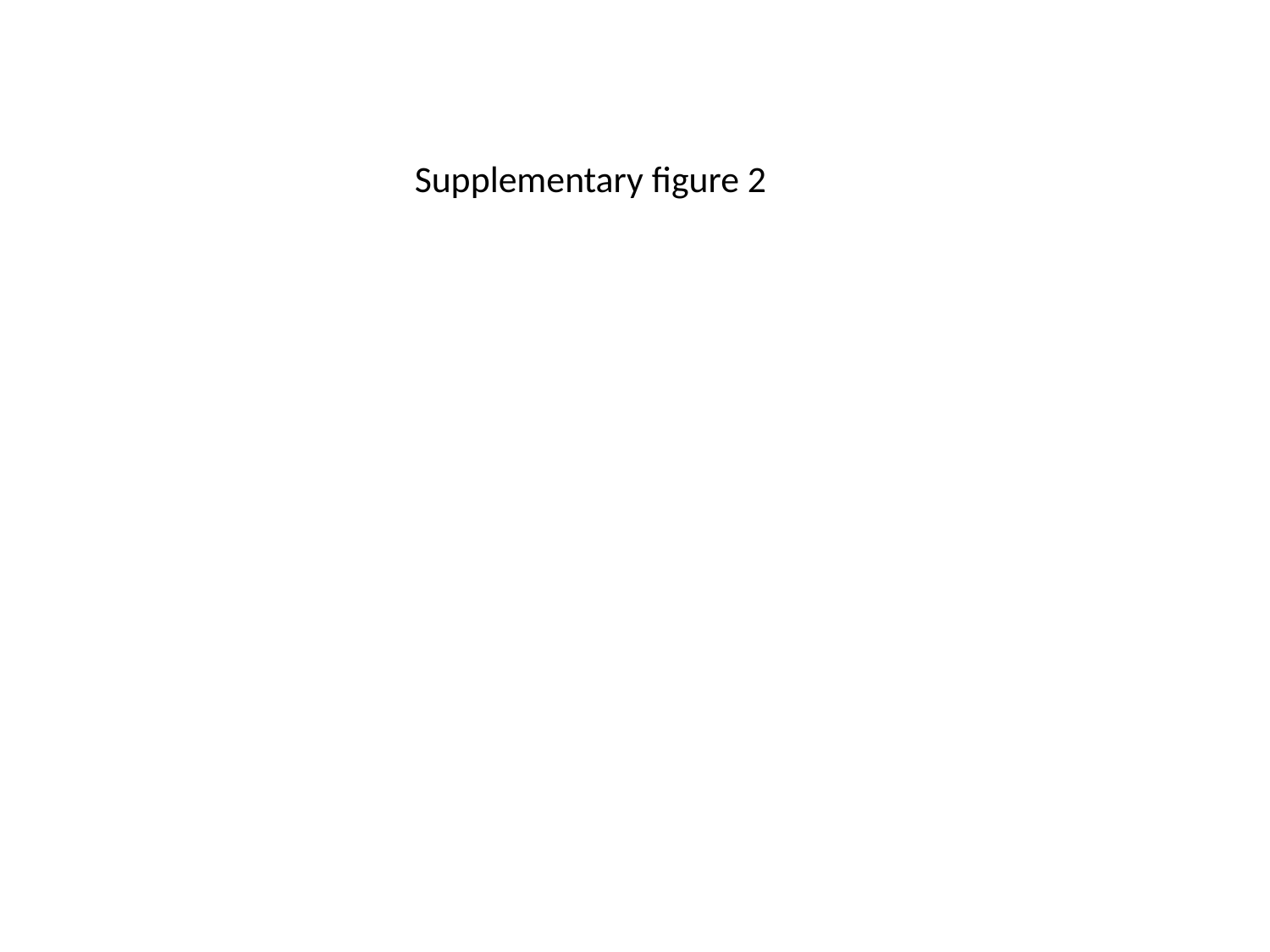

Supplementary figure 2

## Slide 2
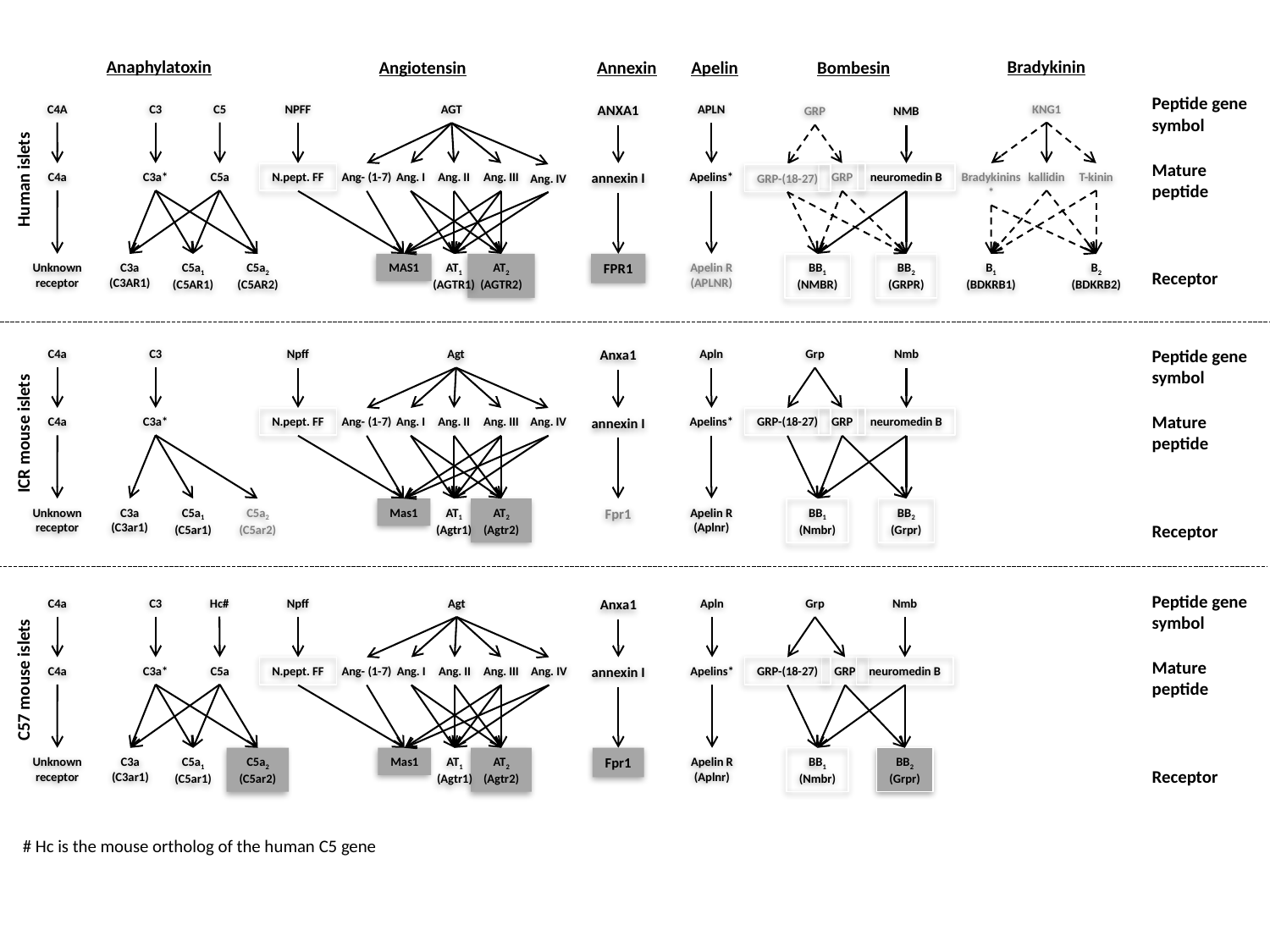

Annexin
ANXA1
annexin I
FPR1
Anxa1
annexin I
Fpr1
Anxa1
annexin I
Fpr1
Angiotensin
NPFF
AGT
N.pept. FF
Ang- (1-7)
Ang. I
Ang. II
Ang. III
Ang. IV
MAS1
AT1
(AGTR1)
AT2
(AGTR2)
Npff
Agt
N.pept. FF
Ang- (1-7)
Ang. I
Ang. II
Ang. III
Ang. IV
Mas1
AT1
(Agtr1)
AT2
(Agtr2)
Npff
Agt
N.pept. FF
Ang- (1-7)
Ang. I
Ang. II
Ang. III
Ang. IV
AT2
(Agtr2)
Mas1
AT1
(Agtr1)
Apelin
APLN
Apelins*
Apelin R
(APLNR)
Apln
Apelins*
Apelin R
(Aplnr)
Apln
Apelins*
Apelin R
(Aplnr)
Anaphylatoxin
Bombesin
GRP
NMB
GRP
neuromedin B
GRP-(18-27)
BB1
(NMBR)
BB2
(GRPR)
Grp
Nmb
GRP
neuromedin B
GRP-(18-27)
BB1
(Nmbr)
BB2
(Grpr)
Grp
Nmb
GRP-(18-27)
GRP
neuromedin B
BB1
(Nmbr)
BB2
(Grpr)
Bradykinin
Peptide gene symbol
Mature peptide
Receptor
Human islets
ICR mouse islets
C57 mouse islets
Peptide gene symbol
Mature peptide
Receptor
Peptide gene symbol
Mature peptide
Receptor
KNG1
Bradykinins*
kallidin
T-kinin
B1
(BDKRB1)
B2
(BDKRB2)
C4A
C3
C5
C4a
C3a*
C5a
Unknown
receptor
C3a
(C3AR1)
C5a1
(C5AR1)
C5a2
(C5AR2)
C4a
C3
C4a
C3a*
Unknown
receptor
C3a
(C3ar1)
C5a1
(C5ar1)
C5a2
(C5ar2)
C3
C4a
Hc#
C5a
C4a
C3a*
C3a
(C3ar1)
Unknown
receptor
C5a1
(C5ar1)
C5a2
(C5ar2)
# Hc is the mouse ortholog of the human C5 gene

## Slide 3
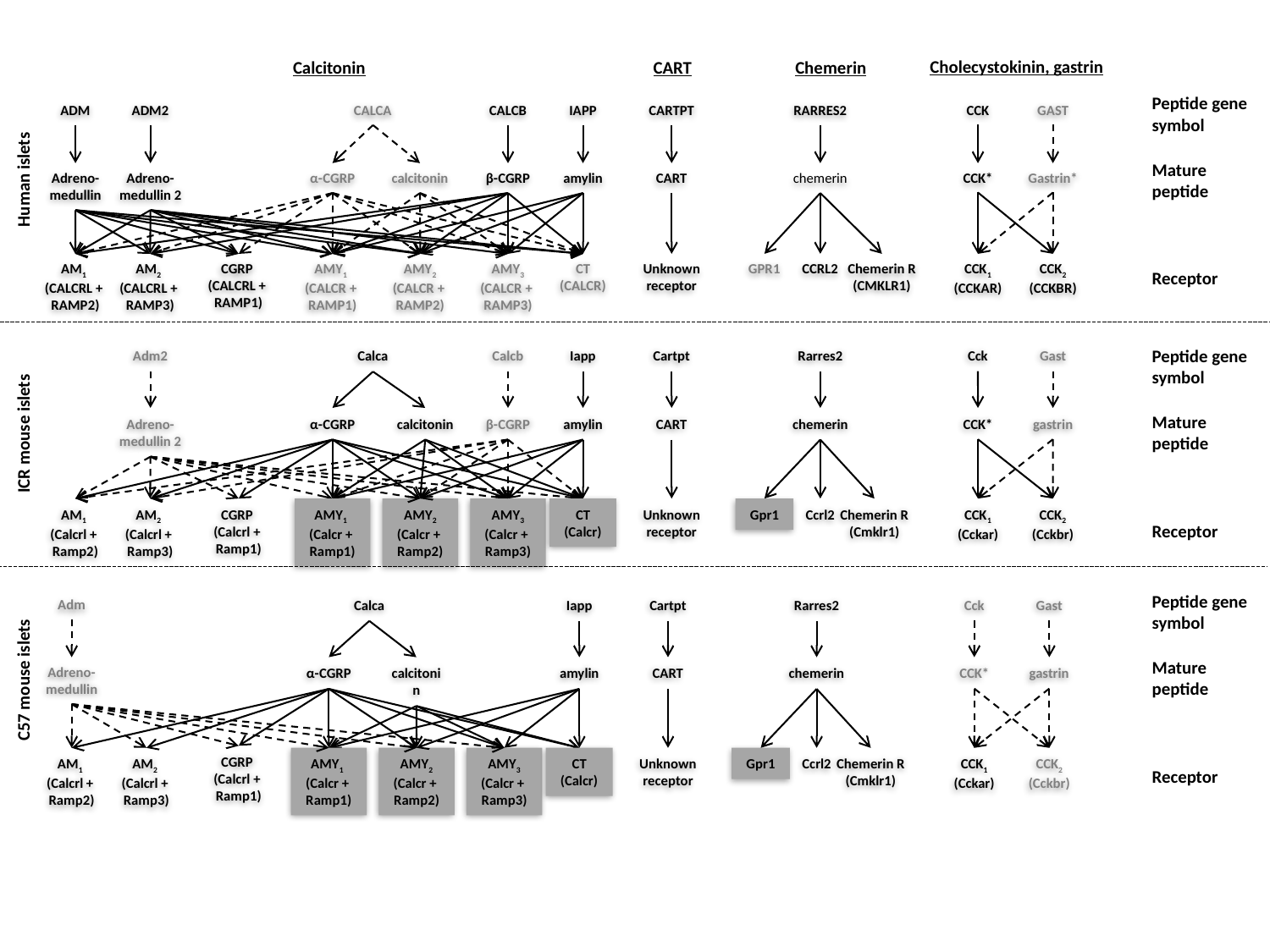

Calcitonin
ADM
ADM2
CALCA
CALCB
IAPP
Adreno-
medullin
Adreno-
medullin 2
α-CGRP
calcitonin
β-CGRP
amylin
CGRP
(CALCRL +
RAMP1)
AM1
(CALCRL +
RAMP2)
AM2
(CALCRL +
RAMP3)
AMY1
(CALCR +
RAMP1)
AMY2
(CALCR +
RAMP2)
AMY3
(CALCR +
RAMP3)
CT
(CALCR)
Adm2
Calca
Calcb
Iapp
Adreno-
medullin 2
α-CGRP
calcitonin
β-CGRP
amylin
AM1
(Calcrl +
Ramp2)
AM2
(Calcrl +
Ramp3)
CGRP
(Calcrl +
Ramp1)
AMY1
(Calcr +
Ramp1)
AMY2
(Calcr +
Ramp2)
AMY3
(Calcr +
Ramp3)
CT
(Calcr)
Adm
Calca
Iapp
α-CGRP
calcitonin
amylin
CGRP
(Calcrl +
Ramp1)
AM2
(Calcrl +
Ramp3)
AMY1
(Calcr +
Ramp1)
AMY2
(Calcr +
Ramp2)
AMY3
(Calcr +
Ramp3)
CT
(Calcr)
Adreno-
medullin
AM1
(Calcrl +
Ramp2)
CART
CARTPT
CART
Unknown
receptor
Cartpt
CART
Unknown
receptor
Cartpt
CART
Unknown
receptor
Chemerin
RARRES2
chemerin
GPR1
CCRL2
Chemerin R
(CMKLR1)
Rarres2
chemerin
Gpr1
Ccrl2
Chemerin R
(Cmklr1)
Rarres2
chemerin
Gpr1
Ccrl2
Chemerin R
(Cmklr1)
Cholecystokinin, gastrin
Peptide gene symbol
Mature peptide
Receptor
Human islets
ICR mouse islets
C57 mouse islets
Peptide gene symbol
Mature peptide
Receptor
Peptide gene symbol
Mature peptide
Receptor
CCK
GAST
CCK*
Gastrin*
CCK1
(CCKAR)
CCK2
(CCKBR)
Cck
Gast
CCK*
gastrin
CCK1
(Cckar)
CCK2
(Cckbr)
Gast
Cck
CCK*
gastrin
CCK1
(Cckar)
CCK2
(Cckbr)

## Slide 4
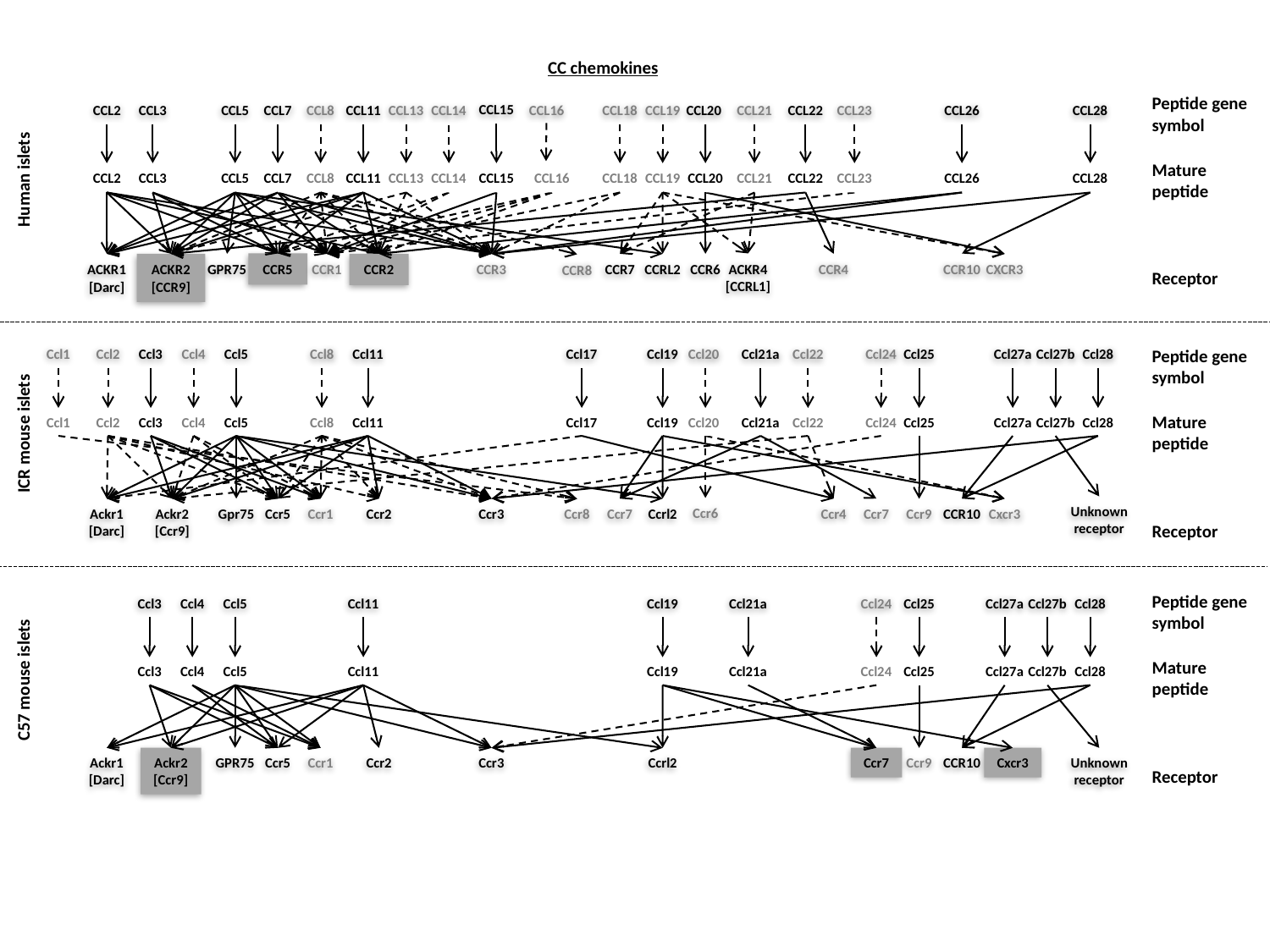

CC chemokines
Peptide gene symbol
Mature peptide
Receptor
Human islets
ICR mouse islets
C57 mouse islets
Peptide gene symbol
Mature peptide
Receptor
Peptide gene symbol
Mature peptide
Receptor
CCL15
CCL2
CCL3
CCL5
CCL7
CCL8
CCL11
CCL13
CCL14
CCL14
CCL16
CCL18
CCL19
CCL20
CCL21
CCL22
CCL23
CCL26
CCL28
CCL2
CCL3
CCL5
CCL7
CCL8
CCL11
CCL13
CCL18
CCL19
CCL20
CCL21
CCL22
CCL23
CCL26
CCL28
CCL15
CCL16
CCR5
ACKR4
[CCRL1]
CCR10
ACKR1
[Darc]
ACKR2
[CCR9]
CCR1
CCR2
CCR3
CCR7
CCRL2
CCR6
CCR4
CXCR3
GPR75
CCR8
Ccl1
Ccl2
Ccl3
Ccl4
Ccl5
Ccl8
Ccl11
Ccl17
Ccl19
Ccl20
Ccl21a
Ccl22
Ccl24
Ccl25
Ccl27a
Ccl27b
Ccl28
Ccl1
Ccl2
Ccl3
Ccl4
Ccl5
Ccl8
Ccl11
Ccl17
Ccl19
Ccl20
Ccl21a
Ccl22
Ccl24
Ccl25
Ccl27a
Ccl27b
Ccl28
Unknown
receptor
Ccr6
Ackr1
[Darc]
Ackr2
[Ccr9]
Gpr75
Ccr5
Ccr1
Ccr2
Ccr3
Ccr7
Ccrl2
Ccr4
Ccr7
CCR10
Cxcr3
Ccr8
Ccr9
Ccl3
Ccl4
Ccl5
Ccl11
Ccl19
Ccl21a
Ccl24
Ccl25
Ccl27a
Ccl27b
Ccl28
Ccl3
Ccl4
Ccl5
Ccl11
Ccl19
Ccl21a
Ccl24
Ccl25
Ccl27a
Ccl27b
Ccl28
Ackr1
[Darc]
Ackr2
[Ccr9]
Unknown
receptor
GPR75
Ccr5
Ccr1
Ccr2
Ccr3
Ccrl2
Ccr7
Ccr9
CCR10
Cxcr3

## Slide 5
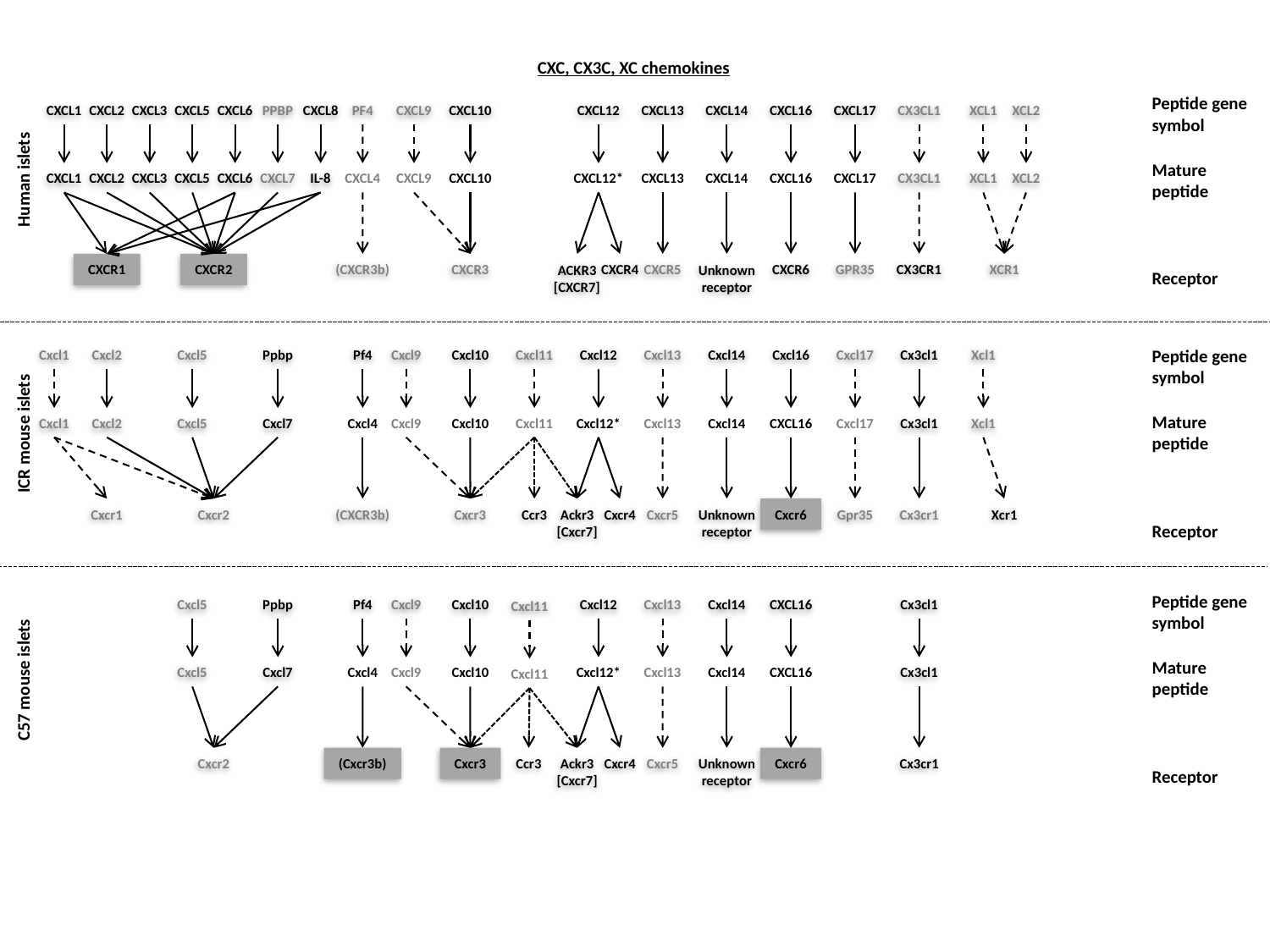

CXC, CX3C, XC chemokines
Peptide gene symbol
Mature peptide
Receptor
Human islets
ICR mouse islets
C57 mouse islets
Peptide gene symbol
Mature peptide
Receptor
Peptide gene symbol
Mature peptide
Receptor
CXCL1
CXCL2
CXCL3
CXCL5
CXCL6
PPBP
CXCL8
PF4
CXCL9
CXCL10
CXCL12
CXCL13
CXCL14
CXCL16
CXCL17
CX3CL1
XCL1
XCL2
CXCL1
CXCL2
CXCL3
CXCL5
CXCL6
CXCL7
IL-8
CXCL4
CXCL9
CXCL10
CXCL12*
CXCL13
CXCL14
CXCL16
CXCL17
CX3CL1
XCL1
XCL2
CXCR1
CXCR2
(CXCR3b)
CXCR3
CXCR4
CXCR5
CXCR6
GPR35
CX3CR1
XCR1
ACKR3
[CXCR7]
Unknown
receptor
Cxcl1
Cxcl11
Cxcl2
Cxcl5
Ppbp
Pf4
Cxcl9
Cxcl10
Cxcl12
Cxcl13
Cxcl14
Cxcl16
Cxcl17
Cx3cl1
Xcl1
Cxcl1
Cxcl11
Cxcl2
Cxcl5
Cxcl7
Cxcl4
Cxcl9
Cxcl10
Cxcl12*
Cxcl13
Cxcl14
CXCL16
Cxcl17
Cx3cl1
Xcl1
Cxcr1
Cxcr2
(CXCR3b)
Cxcr3
Ccr3
Ackr3
[Cxcr7]
Cxcr4
Cxcr5
Unknown
receptor
Cxcr6
Gpr35
Cx3cr1
Xcr1
Cxcl5
Ppbp
Pf4
Cxcl9
Cxcl10
Cxcl12
Cxcl13
Cxcl14
CXCL16
Cx3cl1
Cxcl11
Cxcl5
Cxcl7
Cxcl4
Cxcl9
Cxcl10
Cxcl12*
Cxcl13
Cxcl14
CXCL16
Cx3cl1
Cxcl11
Cxcr2
(Cxcr3b)
Cxcr3
Ackr3
[Cxcr7]
Ccr3
Cxcr4
Cxcr5
Unknown
receptor
Cxcr6
Cx3cr1

## Slide 6
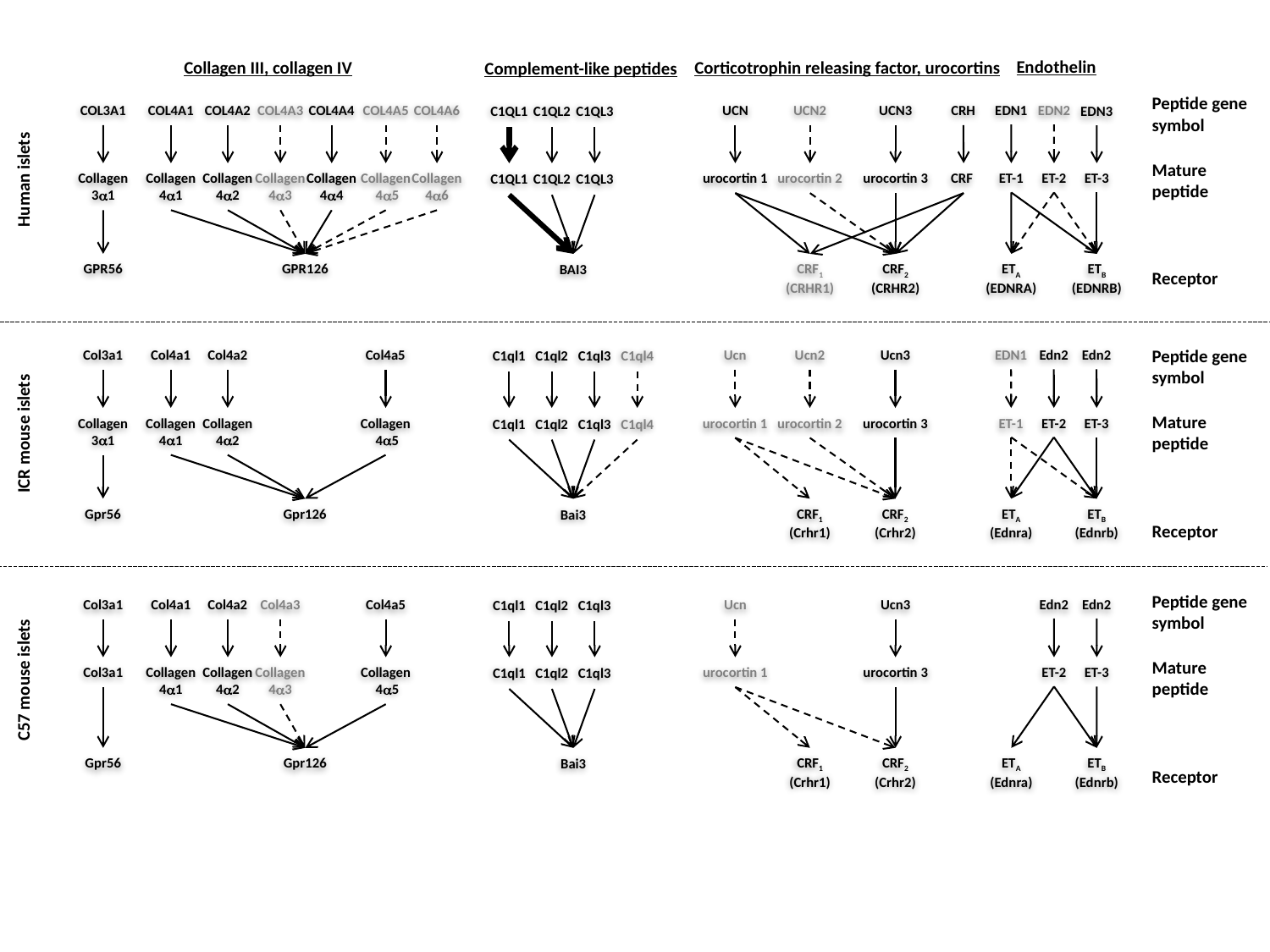

Collagen III, collagen IV
COL3A1
COL4A1
COL4A2
COL4A3
COL4A4
COL4A5
COL4A6
Collagen
3a1
Collagen
4a1
Collagen
4a2
Collagen
4a3
Collagen
4a4
Collagen
 4a5
Collagen
4a6
GPR56
GPR126
Col3a1
Col4a1
Col4a2
Col4a5
Collagen
3a1
Collagen
4a1
Collagen
4a2
Collagen
 4a5
Gpr56
Gpr126
Col3a1
Col4a1
Col4a2
Col4a3
Col4a5
Col3a1
Collagen
4a1
Collagen
4a2
Collagen
4a3
Collagen
 4a5
Gpr56
Gpr126
Corticotrophin releasing factor, urocortins
UCN
UCN2
UCN3
CRH
urocortin 1
urocortin 2
urocortin 3
CRF
CRF1
(CRHR1)
CRF2
(CRHR2)
Ucn
Ucn2
Ucn3
urocortin 1
urocortin 2
urocortin 3
CRF1
(Crhr1)
CRF2
(Crhr2)
Ucn
Ucn3
urocortin 1
urocortin 3
CRF1
(Crhr1)
CRF2
(Crhr2)
Endothelin
Complement-like peptides
C1QL1
C1QL2
C1QL3
C1QL1
C1QL2
C1QL3
BAI3
C1ql1
C1ql2
C1ql3
C1ql4
C1ql1
C1ql2
C1ql3
C1ql4
Bai3
C1ql1
C1ql2
C1ql3
C1ql1
C1ql2
C1ql3
Bai3
Peptide gene symbol
Mature peptide
Receptor
Human islets
ICR mouse islets
C57 mouse islets
Peptide gene symbol
Mature peptide
Receptor
Peptide gene symbol
Mature peptide
Receptor
EDN1
EDN2
EDN3
ET-1
ET-2
ET-3
ETA
(EDNRA)
ETB
(EDNRB)
EDN1
Edn2
Edn2
ET-1
ET-2
ET-3
ETA
(Ednra)
ETB
(Ednrb)
Edn2
Edn2
ET-2
ET-3
ETA
(Ednra)
ETB
(Ednrb)

## Slide 7
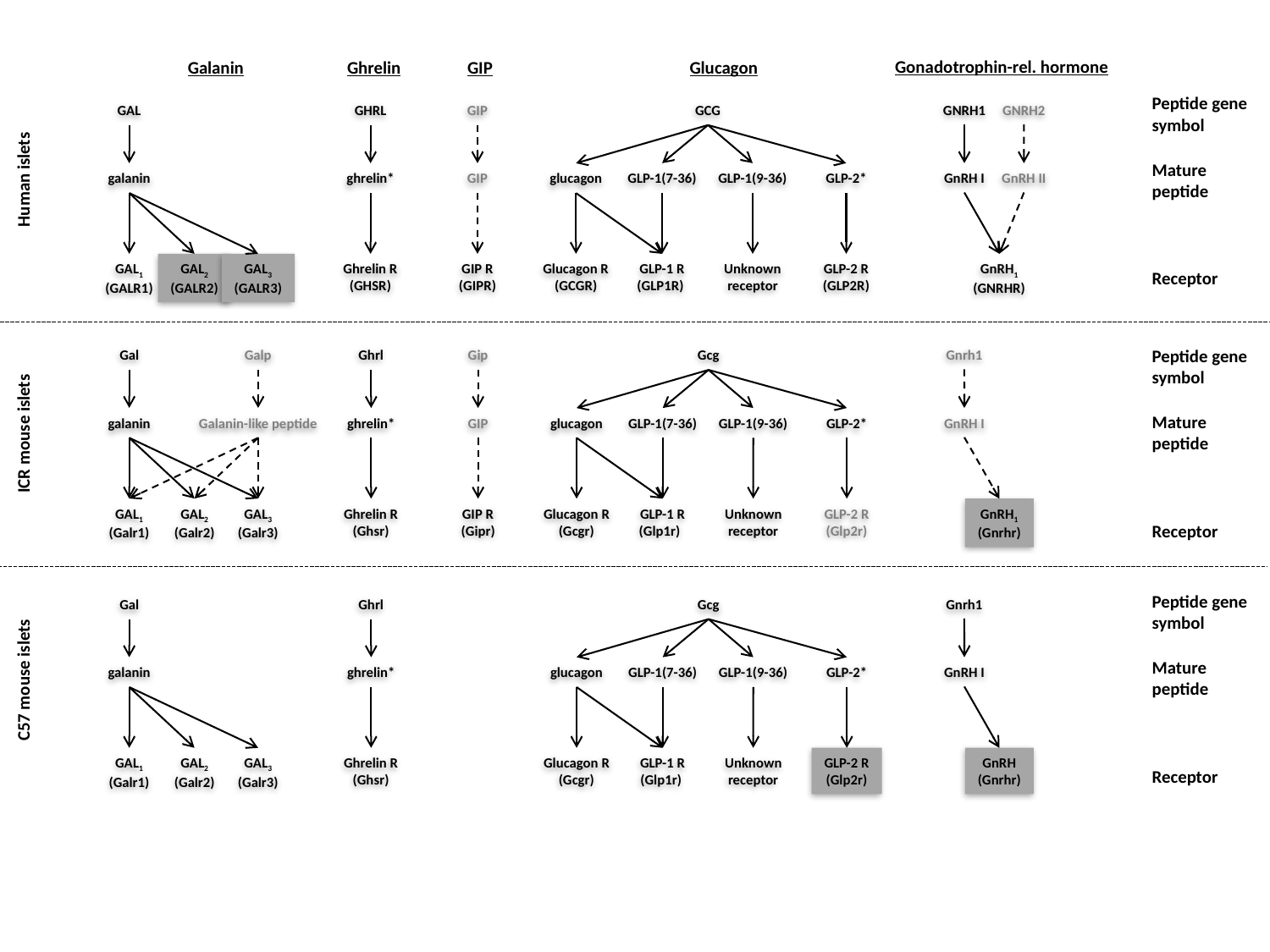

Galanin
GAL
galanin
GAL1
(GALR1)
GAL2
(GALR2)
GAL3
(GALR3)
Gal
Galp
galanin
Galanin-like peptide
GAL1
(Galr1)
GAL2
(Galr2)
GAL3
(Galr3)
Gal
galanin
GAL1
(Galr1)
GAL2
(Galr2)
GAL3
(Galr3)
Ghrelin
GHRL
ghrelin*
Ghrelin R
(GHSR)
Ghrl
ghrelin*
Ghrelin R
(Ghsr)
Ghrl
ghrelin*
Ghrelin R
(Ghsr)
GIP
GIP
GIP
GIP R
(GIPR)
Gip
GIP
GIP R
(Gipr)
Glucagon
GCG
glucagon
GLP-1(7-36)
GLP-1(9-36)
GLP-2*
Glucagon R
(GCGR)
GLP-1 R
(GLP1R)
Unknown
receptor
GLP-2 R
(GLP2R)
Gcg
glucagon
GLP-1(7-36)
GLP-1(9-36)
GLP-2*
Glucagon R
(Gcgr)
GLP-1 R
(Glp1r)
Unknown
receptor
GLP-2 R
(Glp2r)
Gcg
glucagon
GLP-1(7-36)
GLP-1(9-36)
GLP-2*
Glucagon R
(Gcgr)
GLP-1 R
(Glp1r)
Unknown
receptor
GLP-2 R
(Glp2r)
Gonadotrophin-rel. hormone
Peptide gene symbol
Mature peptide
Receptor
Human islets
ICR mouse islets
C57 mouse islets
Peptide gene symbol
Mature peptide
Receptor
Peptide gene symbol
Mature peptide
Receptor
GNRH1
GNRH2
GnRH I
GnRH II
GnRH1
(GNRHR)
Gnrh1
GnRH I
GnRH1
(Gnrhr)
Gnrh1
GnRH I
GnRH
(Gnrhr)

## Slide 8
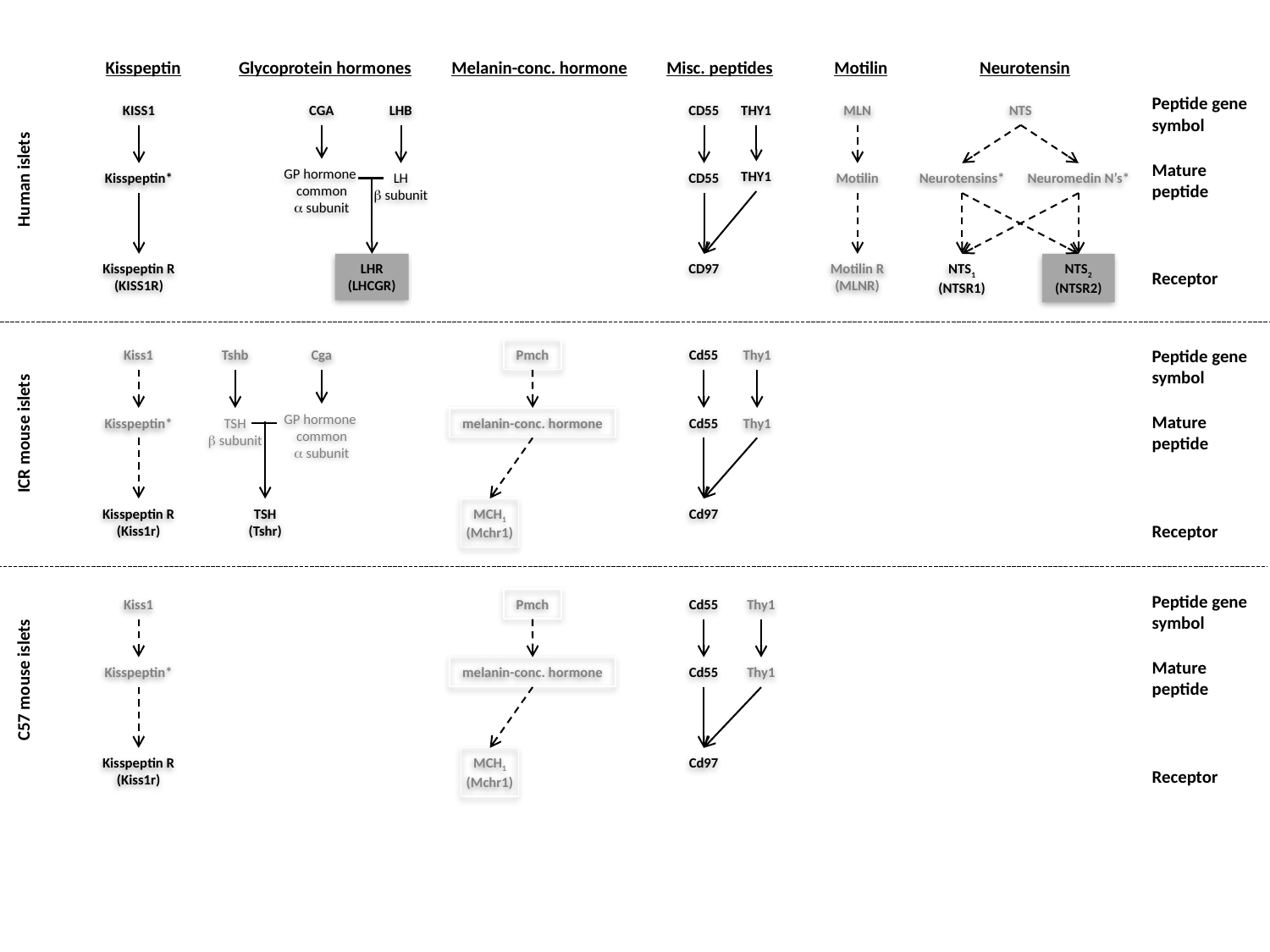

Misc. peptides
CD55
THY1
THY1
CD55
CD97
Cd55
Thy1
Cd55
Thy1
Cd97
Cd55
Thy1
Cd55
Thy1
Cd97
Motilin
MLN
Motilin
Motilin R
(MLNR)
Neurotensin
NTS
Neurotensins*
Neuromedin N’s*
NTS1
(NTSR1)
NTS2
(NTSR2)
Kisspeptin
KISS1
Kisspeptin*
Kisspeptin R
(KISS1R)
Kiss1
Kisspeptin*
Kisspeptin R
(Kiss1r)
Kiss1
Kisspeptin*
Kisspeptin R
(Kiss1r)
Glycoprotein hormones
CGA
LHB
LH
b subunit
GP hormone
common
a subunit
LHR
(LHCGR)
Tshb
TSH
b subunit
Cga
GP hormone
common
a subunit
TSH
(Tshr)
Melanin-conc. hormone
Pmch
melanin-conc. hormone
MCH1
(Mchr1)
Pmch
melanin-conc. hormone
MCH1
(Mchr1)
Peptide gene symbol
Mature peptide
Receptor
Human islets
ICR mouse islets
C57 mouse islets
Peptide gene symbol
Mature peptide
Receptor
Peptide gene symbol
Mature peptide
Receptor

## Slide 9
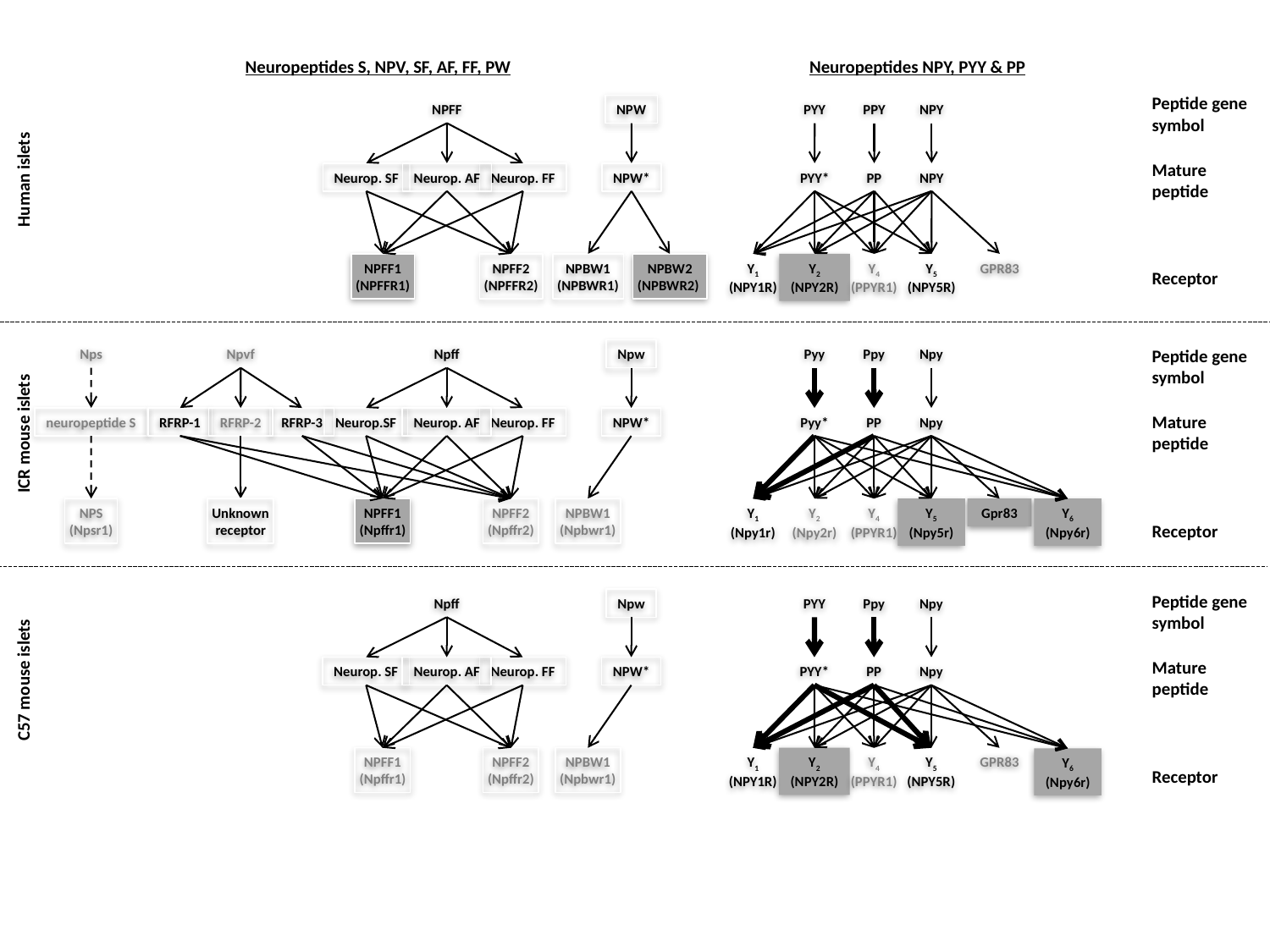

Neuropeptides S, NPV, SF, AF, FF, PW
Neuropeptides NPY, PYY & PP
Peptide gene symbol
Mature peptide
Receptor
Human islets
ICR mouse islets
C57 mouse islets
Peptide gene symbol
Mature peptide
Receptor
Peptide gene symbol
Mature peptide
Receptor
NPFF
NPW
PYY
PPY
NPY
Neurop. SF
Neurop. AF
Neurop. FF
NPW*
PYY*
PP
NPY
NPBW2
(NPBWR2)
Y1
(NPY1R)
Y2
(NPY2R)
Y4
(PPYR1)
Y5
(NPY5R)
GPR83
NPFF1
(NPFFR1)
NPFF2
(NPFFR2)
NPBW1
(NPBWR1)
Nps
Npvf
Npff
Npw
Pyy
Ppy
Npy
neuropeptide S
RFRP-1
RFRP-2
RFRP-3
Neurop.SF
Neurop. AF
Neurop. FF
NPW*
Pyy*
PP
Npy
NPS
(Npsr1)
Unknown
receptor
NPFF1
(Npffr1)
NPFF2
(Npffr2)
NPBW1
(Npbwr1)
Y1
(Npy1r)
Y2
(Npy2r)
Y4
(PPYR1)
Y5
(Npy5r)
Y6
(Npy6r)
Gpr83
Npff
Npw
PYY
Ppy
Npy
Neurop. SF
Neurop. AF
Neurop. FF
NPW*
PYY*
PP
Npy
NPFF1
(Npffr1)
NPFF2
(Npffr2)
Y1
(NPY1R)
Y2
(NPY2R)
Y4
(PPYR1)
Y5
(NPY5R)
GPR83
NPBW1
(Npbwr1)
Y6
(Npy6r)

## Slide 10
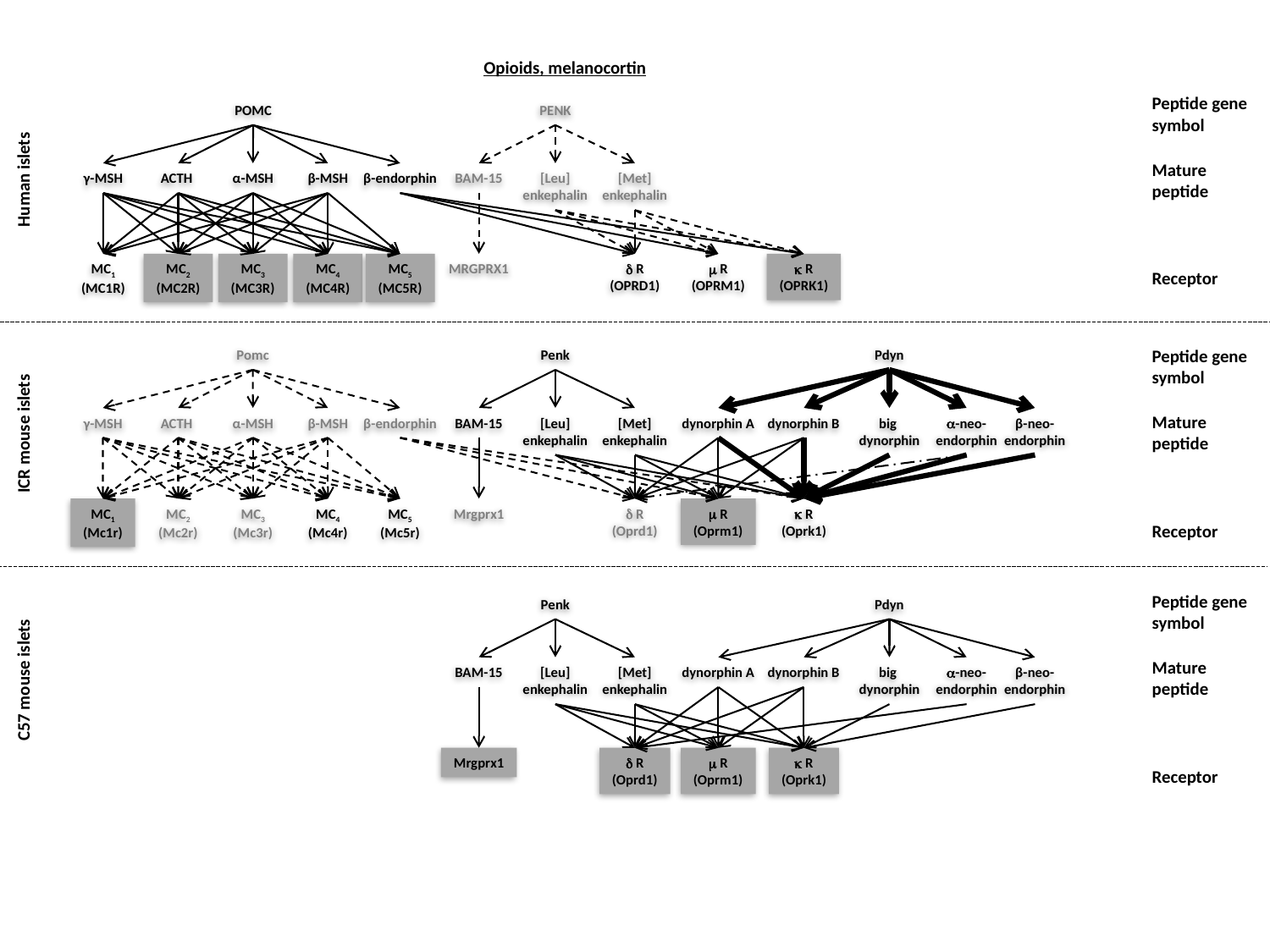

Opioids, melanocortin
POMC
PENK
γ-MSH
ACTH
α-MSH
β-MSH
β-endorphin
BAM-15
[Leu]
enkephalin
[Met]
enkephalin
MC1
(MC1R)
MC2
(MC2R)
MC3
(MC3R)
MC4
(MC4R)
MC5
(MC5R)
MRGPRX1
d R
(OPRD1)
m R
(OPRM1)
k R
(OPRK1)
Pomc
Penk
Pdyn
BAM-15
[Leu]
enkephalin
[Met]
enkephalin
dynorphin A
dynorphin B
big
dynorphin
a-neo-
endorphin
β-neo-
endorphin
γ-MSH
ACTH
α-MSH
β-MSH
β-endorphin
MC1
(Mc1r)
MC2
(Mc2r)
MC3
(Mc3r)
MC4
(Mc4r)
MC5
(Mc5r)
Mrgprx1
d R
(Oprd1)
m R
(Oprm1)
k R
(Oprk1)
Penk
Pdyn
BAM-15
[Leu]
enkephalin
[Met]
enkephalin
dynorphin A
dynorphin B
big
dynorphin
a-neo-
endorphin
β-neo-
endorphin
Mrgprx1
d R
(Oprd1)
m R
(Oprm1)
k R
(Oprk1)
Peptide gene symbol
Mature peptide
Receptor
Human islets
ICR mouse islets
C57 mouse islets
Peptide gene symbol
Mature peptide
Receptor
Peptide gene symbol
Mature peptide
Receptor

## Slide 11
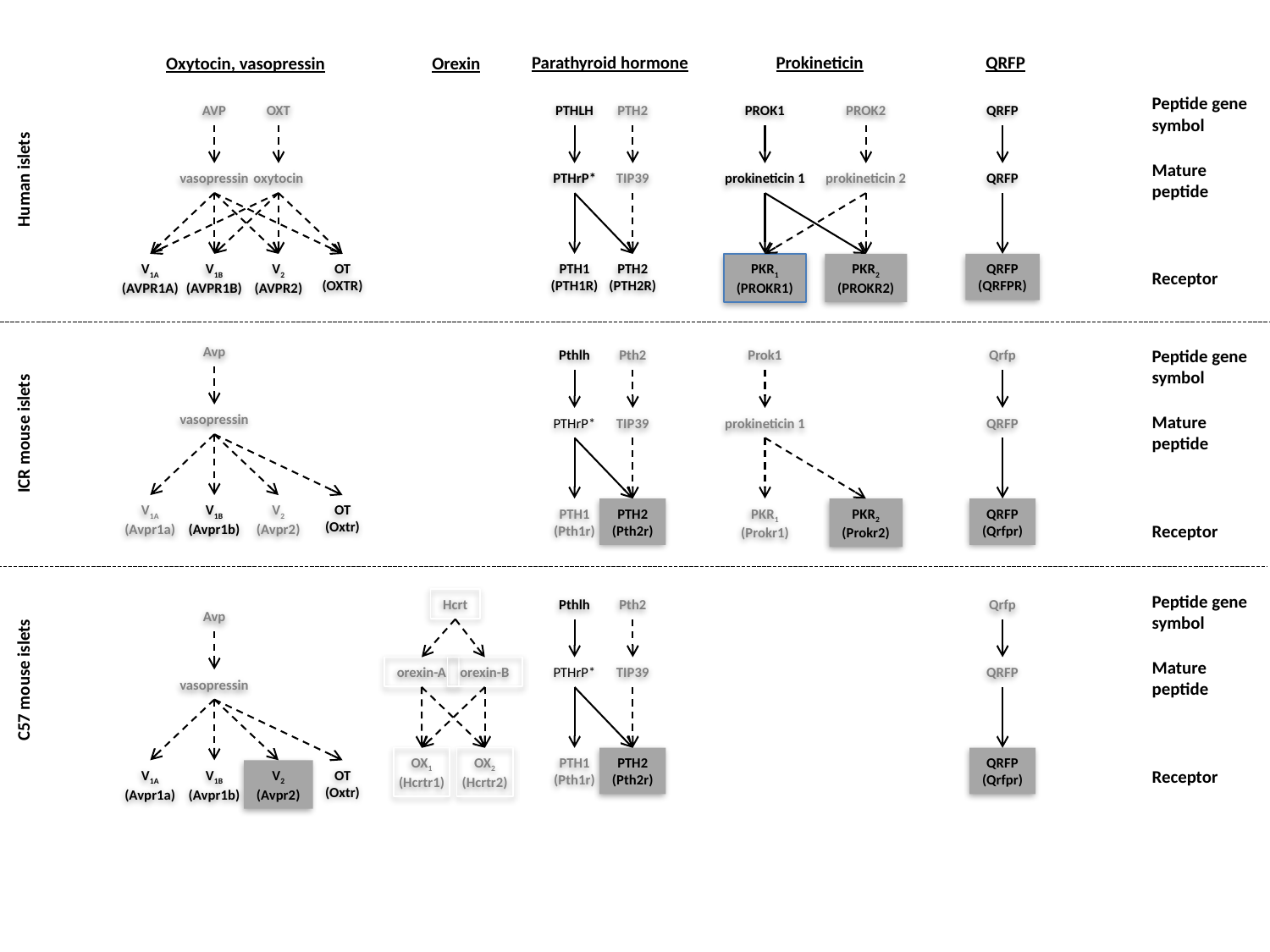

Oxytocin, vasopressin
AVP
OXT
vasopressin
oxytocin
V1A
(AVPR1A)
V1B
(AVPR1B)
V2
(AVPR2)
OT
(OXTR)
Avp
vasopressin
V1A
(Avpr1a)
V1B
(Avpr1b)
V2
(Avpr2)
OT
(Oxtr)
Avp
vasopressin
V1A
(Avpr1a)
V1B
(Avpr1b)
V2
(Avpr2)
OT
(Oxtr)
Orexin
Hcrt
orexin-A
orexin-B
OX1
(Hcrtr1)
OX2
(Hcrtr2)
Parathyroid hormone
PTHLH
PTH2
PTHrP*
TIP39
PTH1
(PTH1R)
PTH2
(PTH2R)
Pthlh
Pth2
PTHrP*
TIP39
PTH1
(Pth1r)
PTH2
(Pth2r)
Pthlh
Pth2
TIP39
PTHrP*
PTH1
(Pth1r)
PTH2
(Pth2r)
Prokineticin
PROK1
PROK2
prokineticin 1
prokineticin 2
PKR1
(PROKR1)
PKR2
(PROKR2)
Prok1
prokineticin 1
PKR2
(Prokr2)
PKR1
(Prokr1)
QRFP
QRFP
QRFP
QRFP
(QRFPR)
Qrfp
QRFP
QRFP
(Qrfpr)
Qrfp
QRFP
QRFP
(Qrfpr)
Peptide gene symbol
Mature peptide
Receptor
Human islets
ICR mouse islets
C57 mouse islets
Peptide gene symbol
Mature peptide
Receptor
Peptide gene symbol
Mature peptide
Receptor

## Slide 12
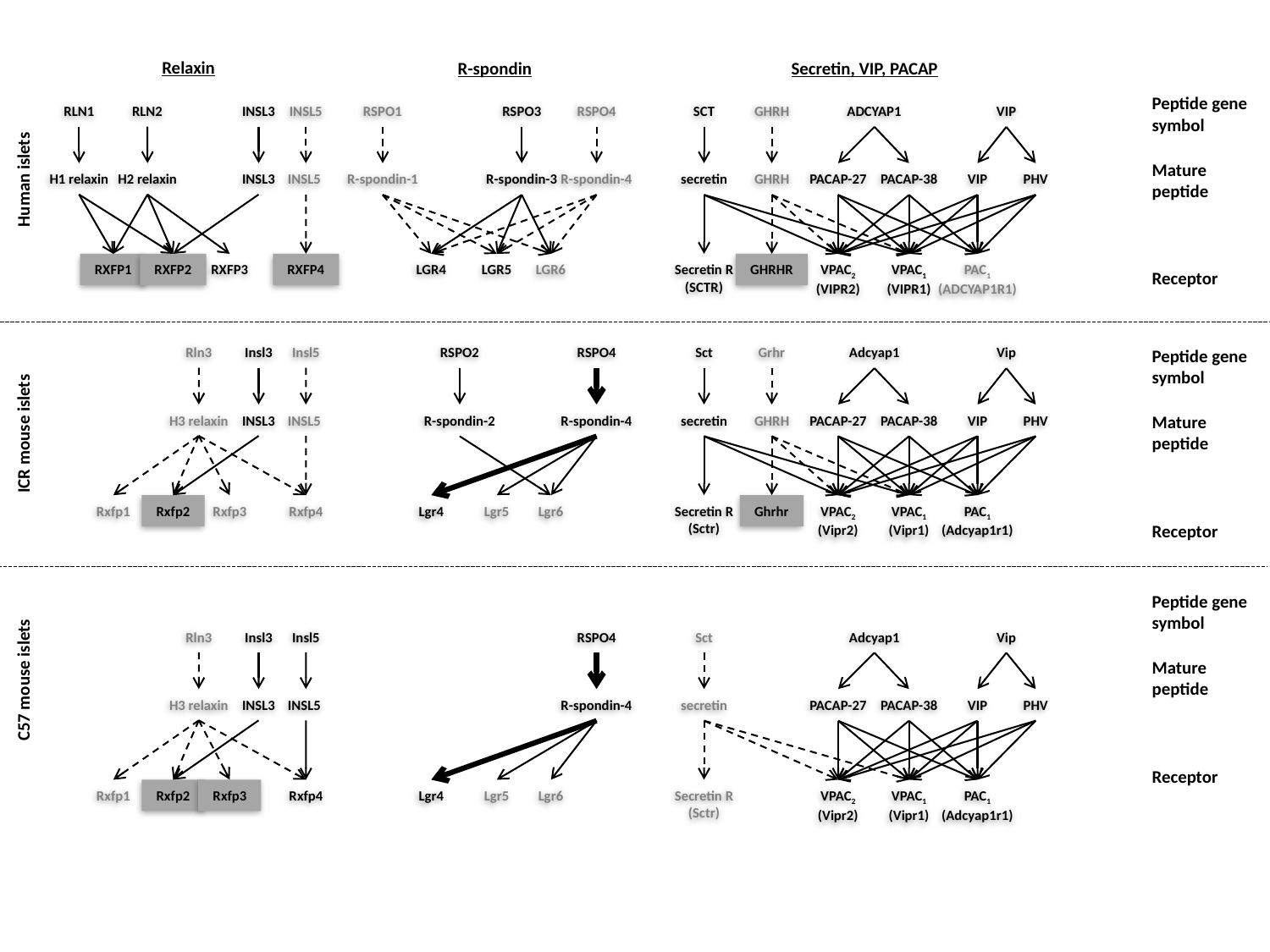

Secretin, VIP, PACAP
SCT
GHRH
ADCYAP1
VIP
secretin
GHRH
PACAP-27
PACAP-38
VIP
PHV
Secretin R
(SCTR)
GHRHR
VPAC2
(VIPR2)
VPAC1
(VIPR1)
PAC1
(ADCYAP1R1)
Sct
Grhr
Adcyap1
Vip
secretin
GHRH
PACAP-27
PACAP-38
VIP
PHV
Secretin R
(Sctr)
Ghrhr
VPAC2
(Vipr2)
VPAC1
(Vipr1)
PAC1
(Adcyap1r1)
Sct
Adcyap1
Vip
PACAP-27
PACAP-38
secretin
VIP
PHV
Secretin R
(Sctr)
VPAC2
(Vipr2)
VPAC1
(Vipr1)
PAC1
(Adcyap1r1)
Relaxin
R-spondin
RSPO1
RSPO3
RSPO4
R-spondin-1
R-spondin-3
R-spondin-4
LGR4
LGR5
LGR6
RSPO2
RSPO4
R-spondin-2
R-spondin-4
Lgr4
Lgr5
Lgr6
RSPO4
R-spondin-4
Lgr4
Lgr5
Lgr6
Peptide gene symbol
Mature peptide
Receptor
Human islets
ICR mouse islets
C57 mouse islets
Peptide gene symbol
Mature peptide
Receptor
Peptide gene symbol
Mature peptide
Receptor
RLN1
RLN2
INSL3
INSL5
H1 relaxin
H2 relaxin
INSL3
INSL5
RXFP1
RXFP2
RXFP3
RXFP4
Rln3
Insl3
Insl5
H3 relaxin
INSL3
INSL5
Rxfp1
Rxfp2
Rxfp3
Rxfp4
Rln3
Insl3
Insl5
H3 relaxin
INSL3
INSL5
Rxfp1
Rxfp2
Rxfp3
Rxfp4

## Slide 13
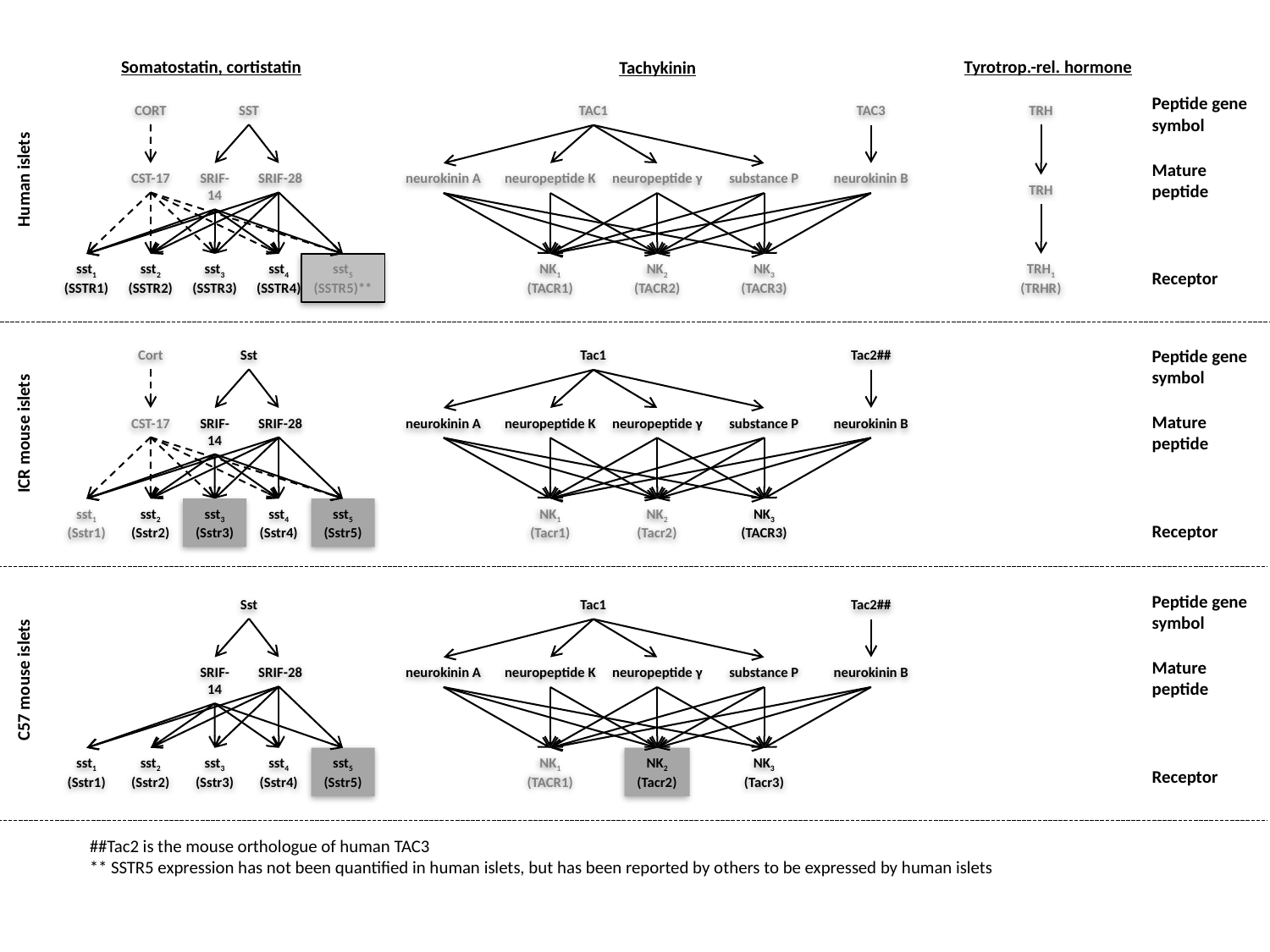

Somatostatin, cortistatin
Tachykinin
TAC1
TAC3
neurokinin A
neuropeptide K
neuropeptide γ
substance P
neurokinin B
NK1
(TACR1)
NK2
(TACR2)
NK3
(TACR3)
Tac1
Tac2##
neurokinin A
neuropeptide K
neuropeptide γ
substance P
neurokinin B
NK1
(Tacr1)
NK2
(Tacr2)
NK3
(TACR3)
Tac1
Tac2##
neurokinin A
neuropeptide K
neuropeptide γ
substance P
neurokinin B
NK1
(TACR1)
NK2
(Tacr2)
NK3
(Tacr3)
Tyrotrop.-rel. hormone
Peptide gene symbol
Mature peptide
Receptor
Human islets
ICR mouse islets
C57 mouse islets
Peptide gene symbol
Mature peptide
Receptor
Peptide gene symbol
Mature peptide
Receptor
TRH
TRH
TRH1
(TRHR)
CORT
SST
CST-17
SRIF-14
 SRIF-28
sst1
(SSTR1)
sst2
(SSTR2)
sst3
(SSTR3)
sst4
(SSTR4)
sst5
(SSTR5)**
Cort
Sst
CST-17
SRIF-14
 SRIF-28
sst1
(Sstr1)
sst2
(Sstr2)
sst3
(Sstr3)
sst4
(Sstr4)
sst5
(Sstr5)
Sst
SRIF-14
 SRIF-28
sst1
(Sstr1)
sst2
(Sstr2)
sst3
(Sstr3)
sst4
(Sstr4)
sst5
(Sstr5)
##Tac2 is the mouse orthologue of human TAC3
** SSTR5 expression has not been quantified in human islets, but has been reported by others to be expressed by human islets

## Slide 14
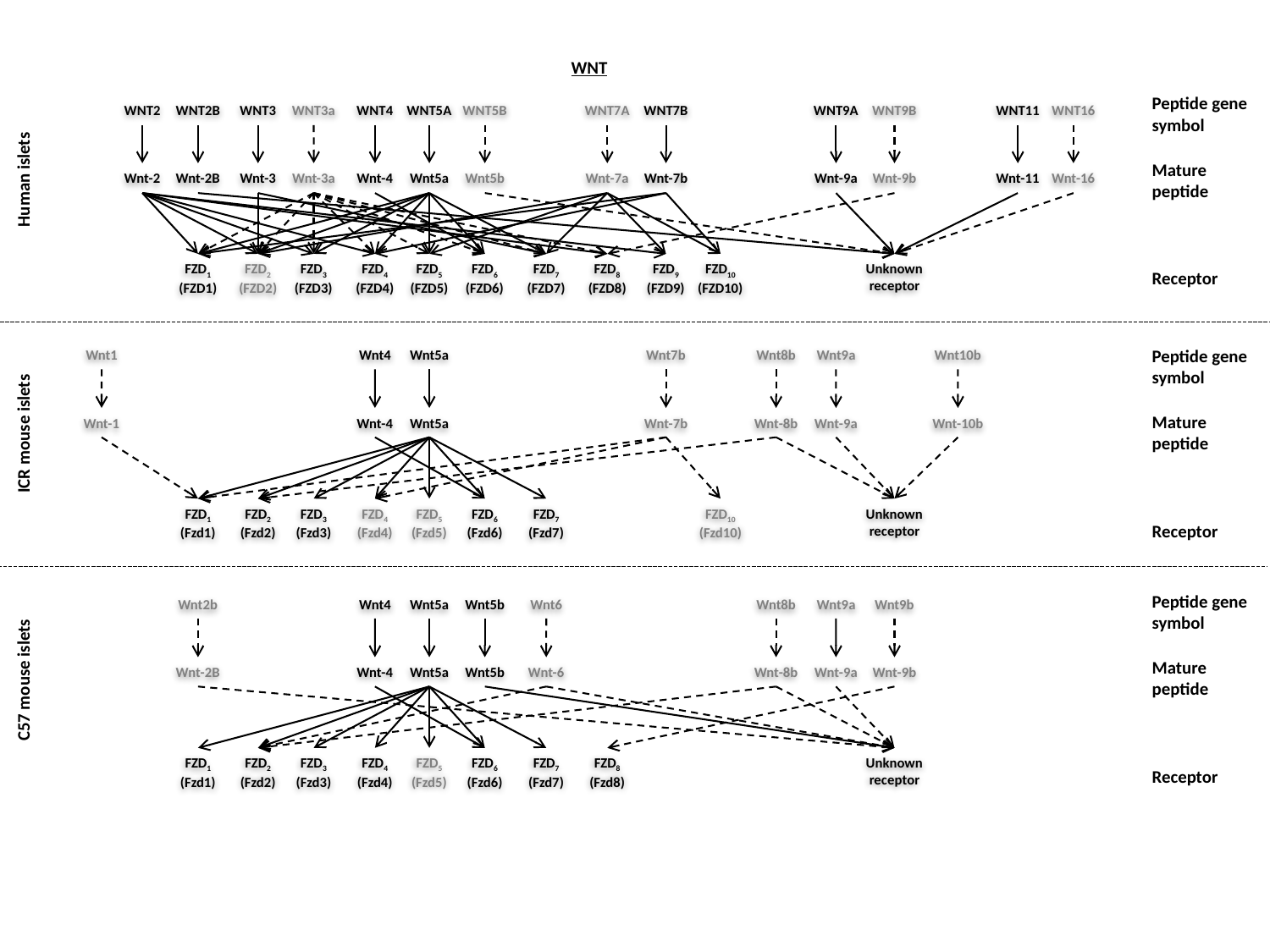

WNT
WNT2
WNT2B
WNT3
WNT3a
WNT4
WNT5A
WNT5B
WNT7A
WNT7B
WNT9A
WNT9B
WNT11
WNT16
Wnt-2
Wnt-2B
Wnt-3
Wnt-3a
Wnt-4
Wnt5a
Wnt5b
Wnt-7a
Wnt-7b
Wnt-9a
Wnt-9b
Wnt-11
Wnt-16
FZD1
(FZD1)
FZD2
(FZD2)
FZD3
(FZD3)
FZD4
(FZD4)
FZD5
(FZD5)
FZD6
(FZD6)
FZD7
(FZD7)
FZD8
(FZD8)
FZD9
(FZD9)
FZD10
(FZD10)
Unknown
receptor
Wnt1
Wnt4
Wnt5a
Wnt7b
Wnt8b
Wnt9a
Wnt10b
Wnt-1
Wnt-4
Wnt5a
Wnt-7b
Wnt-8b
Wnt-9a
Wnt-10b
FZD1
(Fzd1)
FZD2
(Fzd2)
FZD3
(Fzd3)
FZD4
(Fzd4)
FZD5
(Fzd5)
FZD6
(Fzd6)
FZD7
(Fzd7)
FZD10
(Fzd10)
Unknown
receptor
Wnt2b
Wnt4
Wnt5a
Wnt5b
Wnt6
Wnt8b
Wnt9a
Wnt9b
Wnt-2B
Wnt-4
Wnt5a
Wnt5b
Wnt-6
Wnt-8b
Wnt-9a
Wnt-9b
FZD1
(Fzd1)
FZD2
(Fzd2)
FZD3
(Fzd3)
FZD4
(Fzd4)
FZD5
(Fzd5)
FZD6
(Fzd6)
FZD7
(Fzd7)
FZD8
(Fzd8)
Unknown
receptor
Peptide gene symbol
Mature peptide
Receptor
Human islets
ICR mouse islets
C57 mouse islets
Peptide gene symbol
Mature peptide
Receptor
Peptide gene symbol
Mature peptide
Receptor
